# Susceptibility to prosocial and antisocial influence in adolescence following mindfulness training

**DOI:** 10.1002/icd.2386

**Published:** 2022-12-04

**Authors:** Jovita T. Leung, Blanca Piera Pi‐Sunyer, Saz P. Ahmed, Lucy Foulkes, Cait Griffin, Ashok Sakhardande, Marc Bennett, Darren L. Dunning, Kirsty Griffiths, Jenna Parker, Willem Kuyken, J. Mark G. Williams, Tim Dalgleish, Sarah‐Jayne Blakemore

**Affiliations:** ^1^ Institute of Cognitive Neuroscience University College London London UK; ^2^ Department of Psychology University of Cambridge Cambridge UK; ^3^ The Anna Freud Centre London UK; ^4^ Medical Research Council Cognition and Brain Sciences Unit University of Cambridge Cambridge UK; ^5^ Department of Psychiatry University of Oxford Oxford UK

**Keywords:** adolescence, mindfulness, social influence

## Abstract

Mindfulness training programmes have shown to encourage prosocial behaviours and reduce antisocial tendencies in adolescents. However, less is known about whether training affects susceptibility to prosocial and antisocial influence. The current study investigated the effect of mindfulness training (compared with an active control) on self‐reported prosocial and antisocial tendencies and susceptibility to prosocial and antisocial influence. 465 adolescents aged 11–16 years were randomly allocated to one of two training programmes. Pre‐ and post‐training, participants completed a social influence task. Self‐reported likelihood of engaging in prosocial and antisocial behaviours did not change post‐training, and regardless of training group, participants showed a higher propensity for prosocial influence than for antisocial influence. Finally, participants were less influenced by antisocial ratings following both training programmes.

## 1 INTRODUCTION

Adolescence, defined as the period of life between 10 and 24 years (Sawyer et al., 2018), is often associated with heightened susceptibility to social influence. This is the case for both prosocial and antisocial behaviours (Monahan et al., 2009; Sijtsema & Lindenberg, 2018; Van Hoorn et al., 2016). Previous studies report that social influence decreases with age between childhood and adulthood (Ahmed et al., 2020; Foulkes et al., 2018; Knoll et al., 2015, 2017), and that adolescents' susceptibility to social influence might be more pronounced when influenced to behave more positively, relative to being influenced to behave more negatively (Chierchia et al., 2020; Do et al., 2020).

Mindfulness training has shown promising effects in promoting positive behaviour (e.g. helping behaviour) and reducing antisocial tendencies in adolescents (Bögels et al., 2008; Donald et al., 2019; Franco et al., 2016). Mindfulness refers to the regulation of attention to focus on an individual's present moment experiences with a curious and open attitude (Bishop et al., 2004). A recent systematic review of 16 studies found that mindfulness‐based training led to an increase in prosocial behaviours in children and adolescents (Cheang et al., 2019). Mindfulness training can also be efficacious in reducing antisocial behaviours in adolescents (Bögels et al., 2008; Dunning et al., 2019; Franco et al., 2016), with one study finding that a 10‐week mindfulness programme reduced self‐reported aggression in 12–19 year olds relative to a wait list control group. (Franco et al., 2016). However, a meta‐analysis showed that mindfulness did not have a significant impact on negative behaviour (e.g. aggression and hostility) relative to active controls (Dunning et al., 2019). While some of these studies provide evidence that mindfulness training might encourage prosocial behaviours and reduce antisocial behaviours, less is known about how training affects susceptibility to prosocial and antisocial *influence*.

Mindfulness training could modify an individual's susceptibility to prosocial and antisocial influence through its effect on executive control. Specifically, it has been suggested that the benefits of mindfulness might be partly attributable to improved self‐control, that is, the ability to inhibit prepotent responses to effectively respond to goal‐relevant information (Elkins‐Brown et al., 2017; Masicampo & Baumeister, 2007). One study showed that adolescents (13–18 years) with low levels of self‐control were more susceptible to antisocial influence and more likely to become involved with deviant peers (Marshal, Molina & Pelham, 2003). Another large‐scale study found that young adolescents (aged 12–15) with higher levels of self‐control were less susceptible to peer influence (Meldrum et al., 2013).

Moderate correlations (*r* = 0.46) between self‐control and self‐reported mindfulness have been reported in adolescents aged 12–14 years (Riggs et al., 2015). In a study of children aged 9–11 years, higher scores on the mindfulness attention awareness measure were associated with greater accuracy on an inhibitory control task (Oberle et al., 2012). Taken together, these studies suggest that self‐control skills taught during mindfulness training might help to reduce susceptibility to social influence.

### 1.1 The current study

The aim of the current study was to investigate the effect of mindfulness training (versus an active control training programme) on the susceptibility to prosocial and antisocial influence in adolescents. Participants were randomly allocated to an 8‐week programme of mindfulness training or student skills training. Both programmes are active in teaching social, self‐management and cognitive skills such as improving memory. However, mindfulness training is expected to target executive functioning by learning mindfulness skills, where the active control training programme contained no mindfulness skills training (e.g. breathing exercises or reflecting activities were removed from the student skills training; adapted from Student Success Skills; Atlantic Education Consultants, 2013). Therefore, the mindfulness, but not the control training, consisted of activities focusing on breathing, decentring and better focus (MiSP, 2009). Participants completed a social influence task before and after the training. In the task, participants first rated how likely they would be to engage in a prosocial or antisocial behaviour (first‐rating) and were then presented with the average rating for the same behaviour purportedly from other similar‐aged participants (the “provided rating”, which was in fact randomly generated). Participants then re‐rated how likely they would be to engage in the same behaviour (second‐rating). The outcome of interest was the difference between participants' first‐ratings and their second‐ratings after seeing the provided ratings. The greater the difference between the first‐ and second‐ratings, the greater the influence.

In the study protocol, we included the hypothesis that mindfulness training versus the control training would lead to lower susceptibility to social influence from Time 1 to Time 2. Here, we have further specified this hypothesis to better reflect the social influence paradigm used in this study and to align with previous studies that have employed the same paradigm (e.g., Foulkes et al., 2018).

Based on the research described above, we hypothesised that:

1. Self‐reported prosocial behaviour (first‐ratings in the prosocial condition) would increase, and self‐reported antisocial behaviour (first‐ratings in the antisocial condition) would decrease following mindfulness training relative to the active control training.
2. Mindfulness training would be associated with a reduction in social influence such that the change from first‐rating to second‐rating would be smaller post‐training relative to pre‐training.
  - This reduction in social influence would be different across prosocial and antisocial conditions.
  - This reduction in social influence would be greater for mindfulness training than for the active control training.

## 2 METHODS

### 2.1 Participants

**FIGURE 1:**
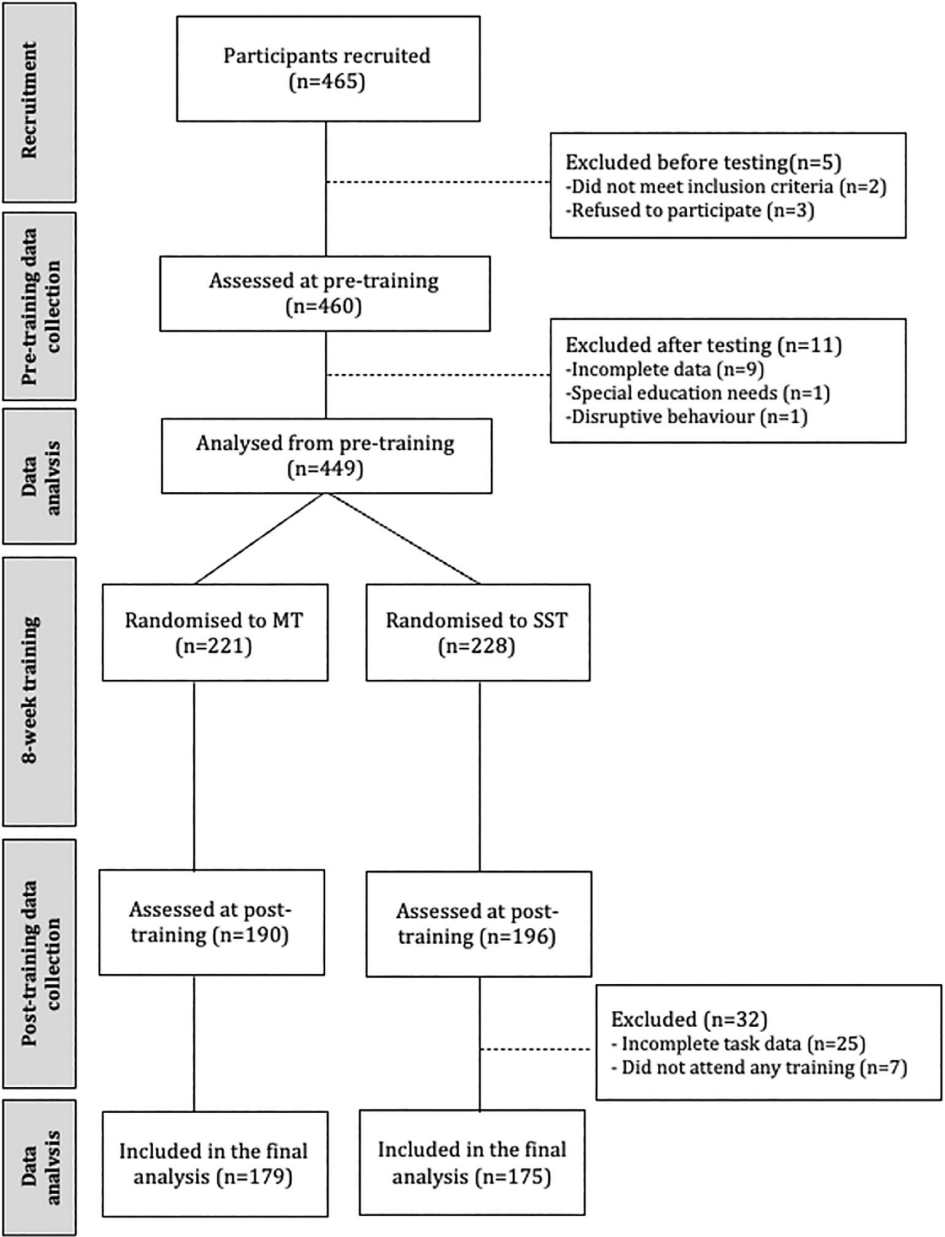
CONSORT diagram showing the flow of participants through each stage of the study. Participants were split into two groups after the pre‐training data collection session; one group received MT and the other group received SST

Participants were recruited as part of a study investigating the mechanisms of change in adolescent mindfulness training (https://osf.io/ax79m). A total of 12 schools from Greater London and Cambridgeshire were recruited (between October 2016 and July 2019). The schools recruited contained a combination of non‐selective, state‐maintained schools (8 mixed and 2 single gender) and selective, independent schools (1 mixed and 1 single gender). All schools received a school assessment rating of “Good” or higher. The mean percentage of students eligible for free school meals (a proxy for low SES; Taylor, 2018) across the 12 schools was 33.75% (range 8.9–62.3), which is slightly higher than national median (29.4%; Department of Health and Department for Education 2017). Special schools, alternative provision settings and schools that teach mindfulness to all students were excluded. Pre‐training data from 449 participants was included in the analysis (299 females; mean age = 13.89 years, SD = 1.38; age range = 11.0–16.5 years) and from 354 participants post‐training (226 females; see Table 1 for participant demographics). See Figure 1 for the flow of participants through each stage of the study. IQ was measured using Cattell's Culture Fair Intelligence test (Institute for Personality and Ability, 1973). The test consists of four types of spatial problems (series completions, odd‐one‐out, matrices, topology), and was completed under timed conditions. Mean IQ across the groups pre‐training was 110.03, SD = 16.81, range = 62–155. The study was approved by the University Research Ethics Committee. Informed consent from parents and assent from participants was obtained. Participants were compensated £15 for each testing session, £5 for attending each training lesson and submitting the corresponding homework sheet, and a bonus £10 for attending six or more training lessons and both testing sessions.

**TABLE 1:** Pre‐training descriptives for participants in each training group. Age, IQ, gender, attendance, and homework completion were included as covariates in the sensitivity analyses (see Appendix S1)

	Mindfulness training	Student skills training	Comparison (*t*‐test)
*N*	228 (76 males)	221 (74 males)	
Mean age in years (SD)	13.88 (1.38)	13.89 (1.39)	*t*(446.34) = 0.06, *p* = 0.951
Age range (years)	11.0–16.4	11.0–16.5	
Mean IQ (SD)	110.47 (17.54)	109.58 (16.07)	*t*(435.94) = −0.55, *p* = 0.581
Mean lesson attendance out of 8 lessons (SD)	6.40 (2.47)	6.41 (2.23)	*t*(444.88) = 0.06, *p* = 0.955
Mean homework completion out of 7 pieces (SD)	5.09 (2.31)	4.55 (2.39)	*t*(445.17) = −2.44, *p* = 0.015

### 2.2 Testing procedure and group randomisation

Testing sessions at both pre‐ and post‐training each lasted 3 hours and took place at the school in small groups (between 7–15 participants; group size at Time 1 and Time 2 is included in the statistical model; see more details in Appendix S1). During these sessions, participants completed the social influence task alongside several other cognitive tasks and questionnaires (see https://osf.io/ax79m). Participants were then randomly assigned to mindfulness training (MT) or the student skills training (SST; see below for training details; see Figure 1). The randomisation was conducted by a statistician independent of the research team and researchers involved in the testing sessions were blind to the training group allocation. To minimise selection bias, participants were not randomised to condition until after they completed the pre‐training data collection. Details of the two groups can be found in Table 1.

### 2.3 Training

#### 2.3.1 Mindfulness training (MT)

MT was an adapted version of .b (dot‐be; MiSP, 2009), which is a 10‐week mindfulness course developed by the Mindfulness in Schools Project in the UK for adolescents aged 11–18 years. The MT curriculum was drawn from mindfulness‐based stress reduction (MBSR; Kabat‐Zinn, 1990) and mindfulness‐based cognitive therapy (MBCT; Segal et al., 2002), with the aim of enabling adolescents to learn mindfulness skills. The curriculum was adapted from a 10‐week course to an 8‐week course to allow the training and the pre‐ and post‐training testing sessions to be completed within a single school term.

There were eight lessons: Playing Attention; Taming the Animal Mind; Recognizing Worry; Being Here Now; Stepping Back; Befriending the Difficult; Taking in The Good; and Pulling it All Together. Each lesson was 45‐mins long and was taught by existing mindfulness teachers who have previously been trained in the MT curriculum. Teachers also attended a two‐day workshop where they received additional training on the MT curriculum used in the study.

#### 2.3.2 Control training: Student skills training (SST)

The SST was an adapted version of Student Success Skills (Atlantic Education Consultants, 2013), an 8‐week course developed in the USA. SST was designed to help students improve on their academic and social performance by focusing on key cognitive, social and self‐management skills. An independent mindfulness instructor reviewed the SST to ensure all elements associated with mindfulness (e.g. breathing exercises or reflecting activities) were removed from the SST. There were eight lessons: Casting Your Net; Get in Formation: Remember Not to Forget; What's the Story; If You've Got Nothing Nice to Say; Together We Can Do So Much; Rewind and Replay Part 1; Rewind & Replay Part 2.

The same teachers (*N* = 13) who delivered the MT curriculum also delivered the SST curriculum. All teachers underwent a 2‐day training course prior to taking part in the study. The workshop covered the following: an introduction to the research study, the evidence supporting each training programme, good research practice, the adaptation to the MT training used in the study and the SST training programme. The workshop was delivered by members of the research team, an independent mindfulness instructor and staff from the Mindfulness in Schools Project (MiSP). All teachers completed a declaration form for any affiliation with MiSP and Student Success Skills (no potential conflict of interest was declared).

Both training curriculums were matched in terms of duration and level of engagement. Training was delivered once a week in groups of 10–13 participants. Each lesson lasted 45 minutes and was delivered in a school classroom, either after school or at an agreed time during the school day. Lessons were taught with presentation slides, which included notes for the teacher, learning objectives and instructions for activities. Participants were asked to complete homework after each lesson for both types of training. Homework sheets were provided each week, tailored to the content of that week's lesson. As part of the homework, participants were required to watch an animated video online and practice the skills learnt during the lesson. Students' training adherence was assessed by their attendance at lessons and the number of homework assignments completed and submitted. The fidelity of the teaching was assessed by recording videos of the lessons. Each training group (10–13 students; training group size included in statistical model; see Appendix S1) had one lesson recorded at random. An independent rater who was trained in teaching the MT and SST curriculums rated the videotapes based on adherence to the key elements of each training lesson (see Appendix S2 for the assessment criteria). MT received 95.4% adherence and SST received a 94.5% adherence rate. See https://osf.io/ax79m for further details regarding the training.

### 2.4 Tasks and measures

#### 2.4.1 Social influence task

**FIGURE 2:**
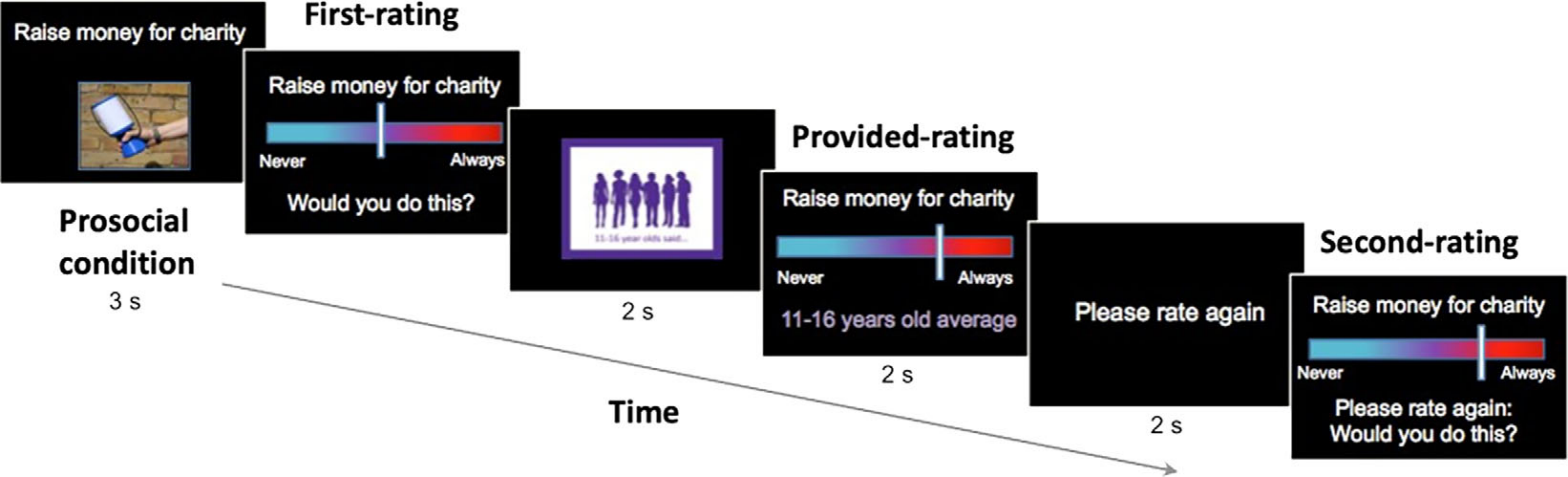
Illustration of the trial sequence in the prosocial condition. Participants were asked to rate how likely they would be to engage in the behaviour (first‐rating). They were then shown the average rating of the 11–16‐year‐olds (provided‐rating) and asked to rate the same scenario again (second‐rating)

The design of the social influence task reported in this study is described in detail in Ahmed et al. (2020) (see Figure 2). Participants were presented with 16 randomly selected scenarios (8 prosocial and 8 antisocial) out of the possible 82, each describing a social behaviour (41 prosocial and 41 antisocial). Prosocial scenarios included helping and sharing behaviours towards friends, family and strangers (e.g., “Give money to charity”, “Help a friend with their schoolwork”). Antisocial scenarios included a range of situations relevant to adolescents, including violation of privacy, indirect and direct aggression, theft and vandalism (e.g., “Make fun of a classmate”, “Talk about a friend behind their back”; see Table S1. for the full list of scenarios).

Participants read the task instructions on the computer screen and completed a practice trial. Each participant then completed 16 trials in a random order. On each trial, participants were shown a short sentence and image depicting either a prosocial or antisocial behaviour (for 3 s; see Figure 2). They were then asked to rate how likely they would be to engage in that behaviour, by using a computer mouse to move a slider to the left side (Never) or to the right side (Always) on a visual analogue scale. The slider first appeared at a random position on the scale to avoid any consistent anchoring bias and there was no time restriction for participants to respond. The position chosen by the participant was recorded to two decimal places as first‐rating (Never = 0.00; Always = 10.00). After making the first rating, participants were shown a rating of the same scenario that was purportedly the average answer provided by other 11–16‐years‐olds (for 2 s). This rating was in fact a randomly generated number between 2 and 8; this range was used to ensure that the number was plausible as an average rating (provided rating). Finally, participants were asked to rate the same scenario again (second‐rating). The task was programmed using Cogent 2000 (University College London Laboratory of Neurobiology; http://www.vislab.ucl.ac.uk/cogent_2000.php), and run in MATLAB (version R2015a; Mathworks Inc., Natick MA). At the end of the second testing session, participants were debriefed and informed that the provided ratings were in fact computer generated.

### 2.5 Statistical analysis

Our analyses included two dependent variables. We first analysed *participants' first‐rating* (Model 1 for Hypothesis 1). First‐ratings ranged from 0 to 10. The second dependent variable was the *change in rating* after observing the ‘provided’ ratings of others (second‐rating – first‐rating; Model 2 for Hypotheses 2). Change in rating ranged from −10 to 10. A positive change in rating value meant that the participant increased their ratings, whereas a negative change in rating value meant the participant decreased their ratings.

The main predictors of interest for Model 1 and Model 2 were social condition (prosocial, antisocial), testing session (pre‐training, post‐training) and training group (MT, SST). In addition, we expected that the change in rating would vary as a function of the discrepancy between the first‐rating and the provided‐rating, as social influence is proportional to the distance between one's baseline behaviour and the decisions of others (Chierchia et al., 2020; Foulkes et al., 2018; Knoll et al., 2015; Moutoussis et al., 2016). We therefore estimated this discrepancy by calculating a *delta rating* score (i.e. the difference between the provided‐rating and first‐rating) and included this as a main predictor for Model 2. Finally, Model 2 also included the *direction of the delta rating* (higher, lower) to decipher effects of increasing or decreasing prosocial and antisocial influence.

Raw trial‐level data were modelled using linear mixed models with the lme4 package (Bates et al., 2015) in the R programming environment (R Core Team, 2013). Best fitting models for each variable were determined through nested model comparison using the same package. The best fitting Model 1 was one that predicted first‐ratings from social condition only. The best fitting Model 2 was one that predicted change in rating from the interaction between the delta rating score, the direction of the delta rating and the social condition; the interaction between the delta rating score, the social condition and the testing session; as well as main effects and lower‐level interactions. As random effects, both Model 1 and Model 2 clustered data by participant (i.e., as random intercepts) and additionally included maximal random slopes for the within‐subject factors (Barr et al., 2013). Main effects and interactions were inspected using omnibus Type III Wald *χ* ^2^ tests and planned and post‐hoc comparisons were performed using the *emmeans* package (Version 1.6.1; Lenth et al., 2018). Details of nested models and full model syntaxes can be found in Appendix S2.

### 2.6 Sensitivity analyses

We inspected whether the omnibus effects remained in a number of sensitivity analyses including relevant factors through nested model comparisons. These included participants' age, gender, IQ, training attendance, amount of homework completed, testing group size at both pre‐ and post‐training, average training group size and extreme values for both Model 1 and Model 2. In addition, we also ran a sensitivity analysis for Model 2 accounting for participant first‐ratings (more information on all sensitivity analyses in Appendix S1).

## 3 RESULTS

### 3.1 Hypothesis 1: Self‐report prosocial and antisocial behaviours (model 1)

**FIGURE 3:**
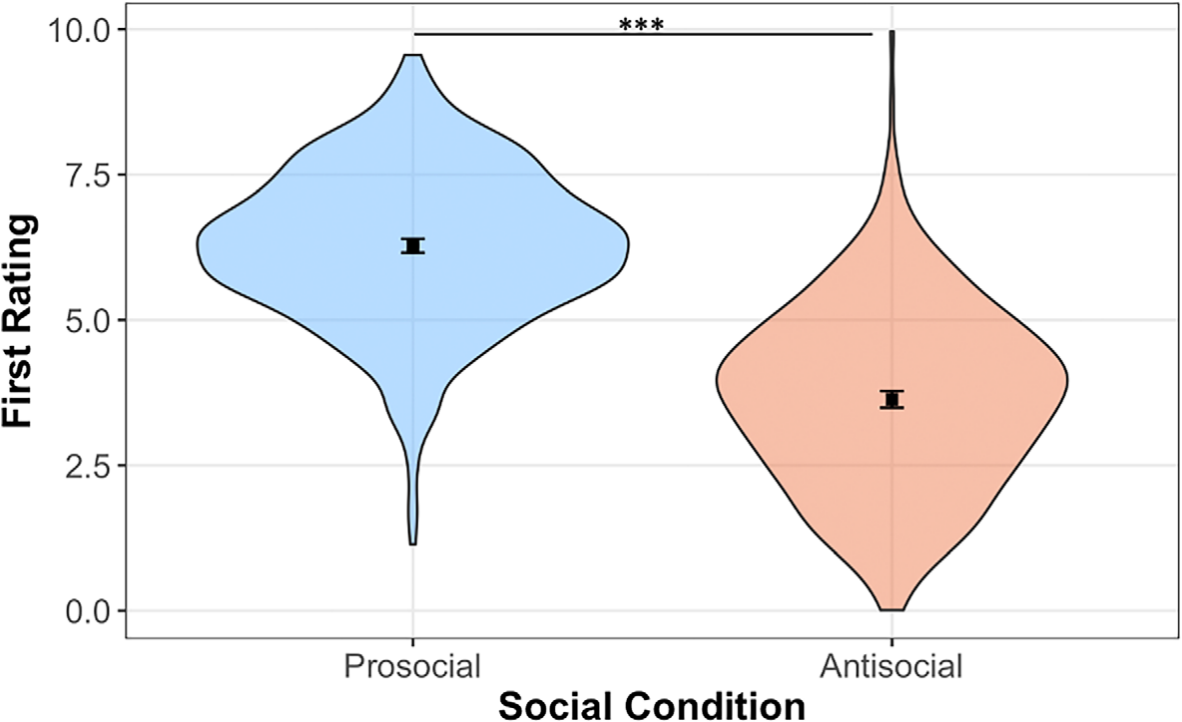
Difference in first‐ratings between social conditions (prosocial, antisocial). The figure shows higher prosocial first‐ratings than antisocial first‐ratings. The violin plots represent kernel probability density of first‐rating values under the prosocial (blue) and antisocial (orange) conditions. Black squares represent the linear mixed model predicted means and error bars show the corresponding 95% intervals. ***p < 0.001

The linear mixed‐effects model revealed a significant main effect of social condition on first‐ratings (*χ* ^2^[1] = 572.33, *p* < 0.001; see Model 1 output in Table S2). Planned contrasts showed that participants produced significantly higher prosocial first‐ratings than antisocial first‐ratings (contrast _Prosocial – Antisocial_ = 2.65, SE = 0.11, *p* < 0.001; Figure 3). Notably, nested model comparisons that included testing session (Δ*χ* ^2^[1] = 0.94, *p* = 0.332) and type of intervention (Δ*χ* ^2^[1] = 0.67, *p* = 0.413) as additional predictors did not fit better than a model omitting these terms. Therefore, contrary to hypothesis 1, our results found no significant differences in first‐ratings post‐training for either the MT or the SST.

### 3.2 Sensitivity analyses

The main effect of social condition on first‐ratings was robust to all sensitivity analyses, including age, gender, IQ, number of lessons attended, amount of homework completed, testing group size pre‐ and post‐training, average training size, as well as after the exclusion of extreme values (all *ps* <0.001, see Table S3 for Model 1 and sensitivity analyses model estimates). A model adjusting for age additionally revealed a significant interaction between age and social condition (*χ* ^2^[1] = 22.96, *p* < 0.001). This was driven by prosocial first‐ratings decreasing with age (slope = −0.10, SE = 0.04, *p* = 0.029), and antisocial first‐ratings increasing with age (slope = 0.28, SE = 0.05, *p* < 0.001, see Figure S1.). In addition, sensitivity analyses showed an interaction between gender and social condition (*χ* ^2^[1] = 8.18, *p* = 0.004). This was driven by females showing higher prosocial first‐ratings (contrast _Female – Male_ = 0.35, SE = 0.12, *p* = 0.007), and lower antisocial first‐ratings (contrast _Female – Male_ = −0.32, SE = 0.13, *p* = 0.041), than males (see Figure S2). For this reason, we additionally account for social condition in the sensitivity analyses for Model 2 when investigating gender and age differences in social influence (see Appendix S1 for more details on sensitivity analyses).

### 3.3 Hypothesis 2: Training effects on social influence (model 2)

**FIGURE 4:**
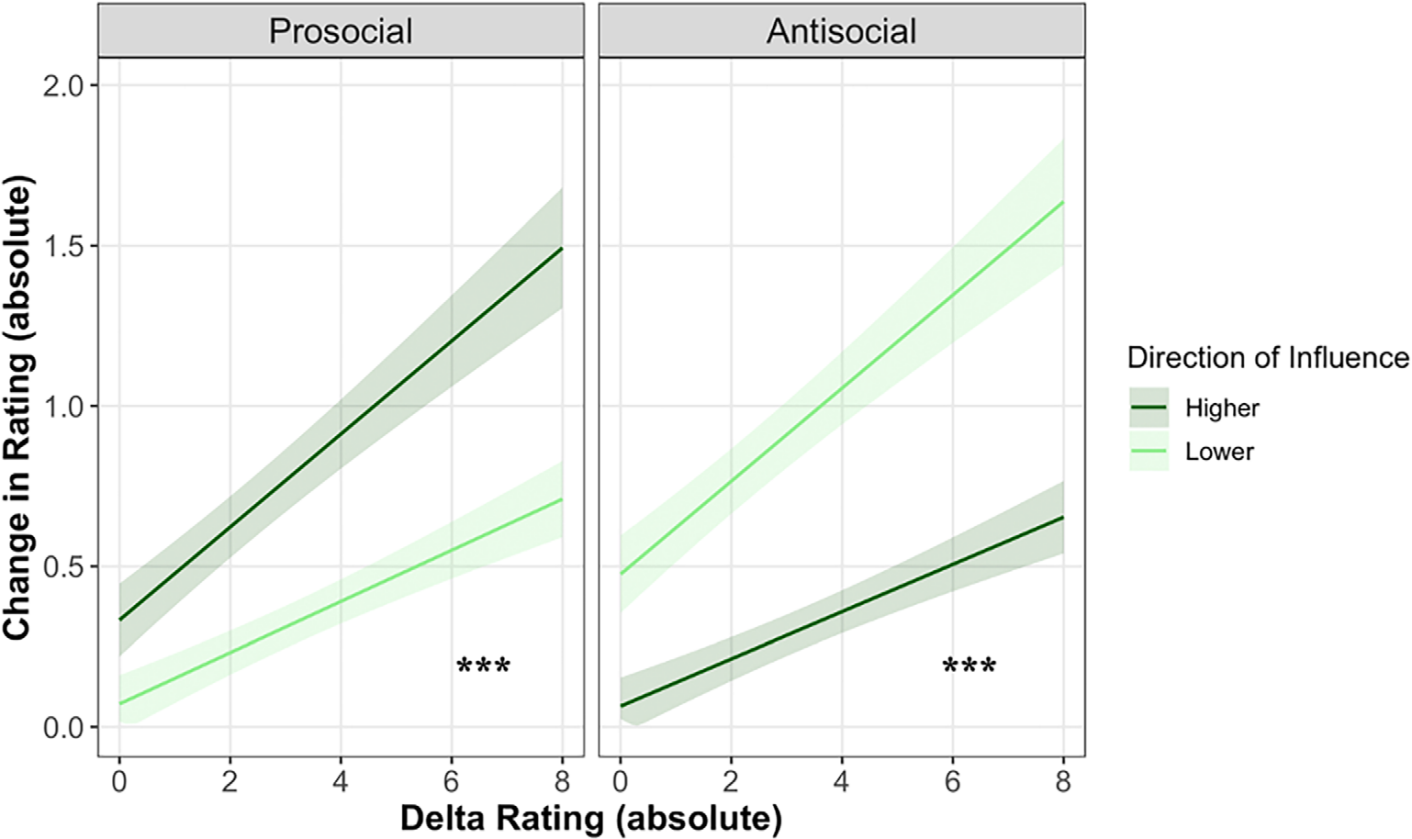
Effect of the direction of social influence (higher, lower) in each social condition (prosocial, antisocial). The plot shows that participants were more socially influenced to increase prosocial ratings rather than to decrease them (left panel), and to decrease antisocial ratings rather than to increase them (right panel). Lines represent the predicted slopes of social influence from higher delta ratings (dark green) and lower delta ratings (light green) and post‐training (dark blue) and shaded areas represent 95% confidence intervals. All values have been converted to absolute terms (i.e., multiplied by −1 if negative) for visualisation purposes. ***p < 0.001

The linear mixed‐effects model revealed a significant main effect of the delta rating on change in rating (*χ* ^2^[1] = 122.64, *p* < 0.001; see Model 2 output in Table S4), such that greater delta ratings were associated with greater change in ratings (slope = 0.15, SE = 0.01, *p* < 0.001). This main effect of delta rating on the change in rating has been previously termed the *social influence effect* (e.g. Foulkes et al., 2018; Knoll et al., 2015; Knoll et al., 2017). In addition, there was a significant three‐way interaction between delta rating, social condition and direction of influence (*χ* ^2^[1] = 36.34, *p* < 0.001; Figure 4). Post hoc comparisons showed that participants were more socially influenced to increase prosocial ratings than to decrease them (contrast _Higher – Lower_ = 0.07, SE = 0.02, *p* < 0.001), as well as to decrease antisocial ratings than to increase them (contrast _Higher – Lower_ = −0.07, SE = 0.02, *p* < 0.001; see all contrast estimates in Table S5). This effect was present across both time points and interventions.

**FIGURE 5:**
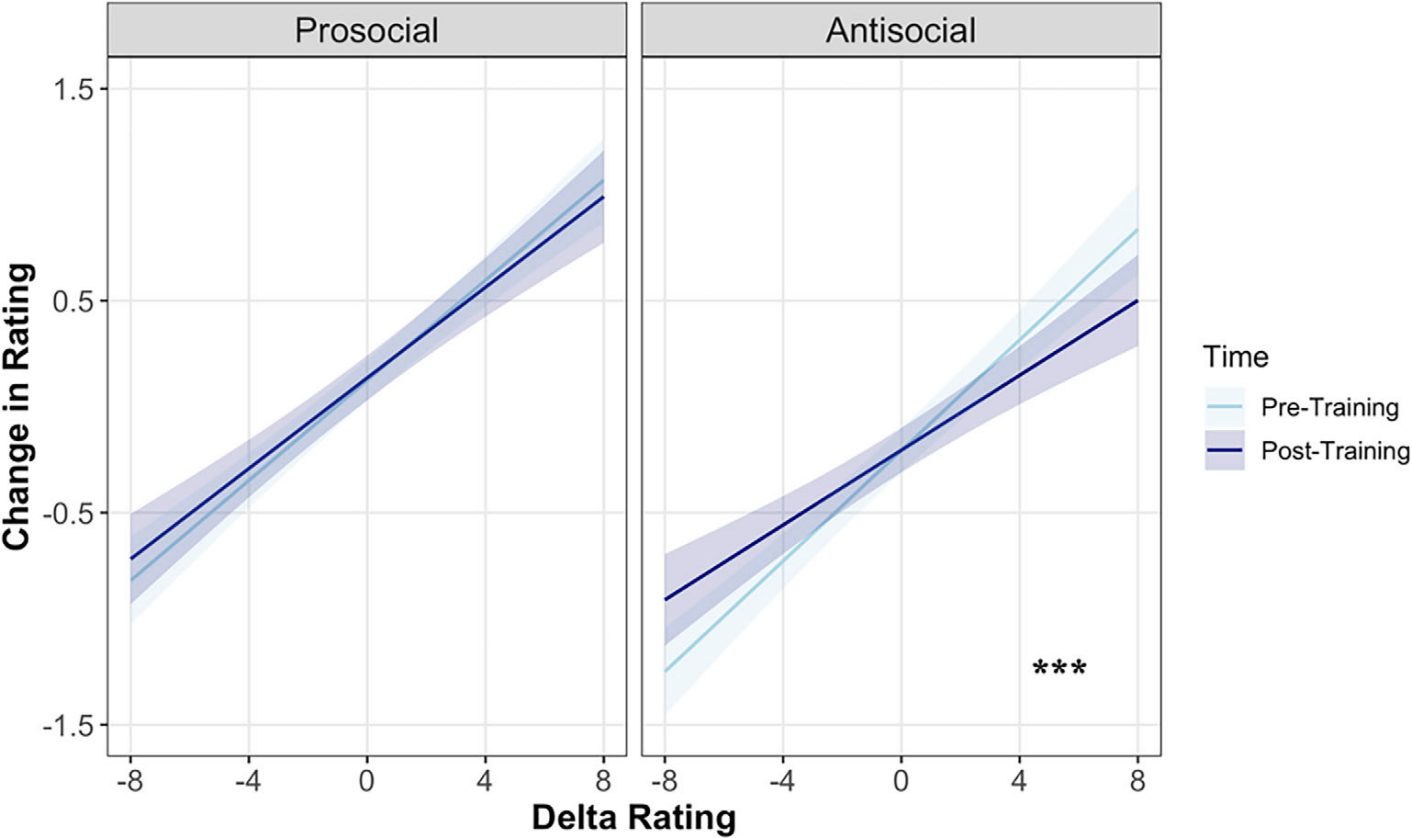
Difference in social influence between testing sessions (pre‐training, post‐training) in each social condition (prosocial, antisocial). The plot shows a significant decrease in social influence at post‐training relative to pre‐training for the antisocial condition (right panel), and no significant difference in social influence pre‐ and post‐training for the prosocial condition (left panel). Lines represent the predicted slopes of social influence pre‐training (light blue) and post‐training (dark blue) and shaded areas represent 95% confidence intervals. ***p < 0.001

Contrary to Hypothesis 2, there was no significant effect of testing session on social influence (*χ* ^2^[1] = 1.36, *p* = 0.243), indicating that there was no overall difference in social influence pre‐ and post‐training, across all conditions. However, there was a significant three‐way interaction between delta rating, social condition and testing session (*χ* ^2^[1] = 6.57, *p* < 0.010; Figure 5). This suggests that there was a difference in social influence pre‐ and post‐training, and that this depended on the specific social condition, supporting hypothesis 2a. Planned contrasts showed that this effect was driven by a decrease in antisocial influence post‐training compared to pre‐training (contrast _Pre‐training – Post‐training_ = 0.04, SE = 0.01, *p* < 0.001), which was not the case for prosocial influence post‐training (contrast _Pre‐training – Post‐training_ = 0.01, SE = 0.01, *p* = 0.243; see all contrast estimates in Table S6).

Nested model comparisons showed that including intervention type as an additional predictor in this model did not fit better than a model omitting this term (Δ*χ* ^2^[2] = 4.10, *p* = 0.129). Therefore, contrary to Hypothesis 2b, the results did not show significant differences between mindfulness training and control training on social influence effects.

### 3.4 Sensitivity analyses

All effects of interest were maintained after adjusting for the relevant factors, including age, gender, IQ, participant first‐ratings, number of lessons attended, amount of homework completed, testing group size pre‐ and post‐training, average training size, as well as after the exclusion of extreme values (see Model 2 and sensitivity analyses model estimates in Table S7). Both the interaction between delta rating, social condition and direction of influence, and the interaction between delta rating, social condition and testing session were robust to all sensitivity analyses. This included models accounting for age and gender differences in social influence between the prosocial and antisocial conditions, both of which were flagged during sensitivity analyses for Model 1 (see relevant section above). These models additionally suggested significant gender differences (*χ*2[1] = 4.87, *p* = 0.027), and this was driven by females being less socially influenced by antisocial ratings than males (contrast _Females – Males_ = − 0.02, SE = 0.01, *p* = 0.022; see Figure S3). There were no significant age differences in social influence between social conditions (*χ*2[1] = 0.01, *p* = 0.933). However, there was an effect of age (*χ*2[1] = 3.90, *p* = 0.049), as well as an effect of IQ (*χ*2[1] = 25.39, *p* < 0.001), on social influence with older participants and participants with a higher IQ being less socially influenced across both social conditions and time points (slope _age_ = − 0.007, SE = 3.54 e^−3^, *p* = 0.049; slope _IQ_ = −0.001, SE = 2.33 e^−4^, *p* < 0.001; see Figures S4 and S5, respectively).

## 4 DISCUSSION

The aim of the current study was to examine whether mindfulness training specifically, compared with an active control, was associated with a change in the self‐reported likelihood of engaging in prosocial and antisocial behaviours and susceptibility to prosocial and antisocial influence. We found that participants' prosocial tendencies were higher than their antisocial tendencies at both time points. In addition, participants were more socially influenced to increase prosocial ratings than to decrease them, and more socially influenced to decrease antisocial ratings than to increase them at both time points. Despite previous studies demonstrating the beneficial effects of mindfulness on adolescents' prosocial and antisocial behaviours (Bögels et al., 2008; Cheang et al., 2019; Donald et al., 2019; Franco et al., 2016), we did not find any significant differences in prosocial or antisocial behaviour (first‐ratings) following an 8‐week mindfulness training programme (or after the control training programme; hypothesis 1). We also found no unique effect of mindfulness (vs. control) training on susceptibility to social influence (hypothesis 2). Instead, participants were less influenced by antisocial, but not prosocial, ratings after *both* training programmes.

Contrary to hypothesis 1, there was no significant change in prosocial and antisocial behaviour following MT, relative to the SST. This is in line with a meta‐analysis that found that, while negative behaviour (e.g. aggression and hostility) was reduced by mindfulness training in studies including a passive control group only, this was not the case for studies that included an active control group (Dunning et al., 2019). However, this meta‐analysis found that younger children showed greater improvements than older children and adolescents following mindfulness‐based interventions (Dunning et al., 2019). The age range included in our sample (11–16 years) was much narrower than the sample included in the meta‐analysis (mean ages ranging from 4.7 to 17.4 years), possibly too narrow to reveal potential age effects in training. Future research should investigate how age influences the effect of mindfulness training on prosocial and antisocial behaviour, using a wider age range than the current sample.

A second aim of the current study was to investigate the impact of mindfulness training on social influence in adolescence. Importantly, before discussing mindfulness training effects on social influence, our results support previously reported findings on social influence during adolescence. To begin, we found that participants revised their ratings to a greater extent as these became increasingly discrepant from observed ratings (i.e. greater delta ratings). This social influence effect is in line with previous studies showing that adolescents are influenced by others' endorsements of prosocial behaviours (Choukas‐Bradley et al., 2015; van Hoorn et al., 2016) and antisocial behaviours (Monahan et al., 2009; Sijtsema & Lindenberg, 2018), and that this social influence effect is stronger when there is a greater disparity between participants' initial responses and others' responses (Chierchia et al., 2020; Foulkes et al., 2018; Knoll et al., 2017). While the social influence effect was still present after accounting for age and IQ, we found that older participants and participants with higher IQ were less susceptible to social influence, which replicates previous findings (Chierchia et al., 2020; Foulkes et al., 2018; Knoll et al., 2015, 2017).

In addition, we also found that the social influence effect was stronger for positive influence than for negative influence. This means that participants were more socially influenced to increase prosocial ratings than to decrease them, as well as to decrease antisocial ratings than to increase them. This suggests that adolescents conform to a greater extent to become more prosocial than more antisocial when presented with information about other people's ratings. This finding builds on recent work suggesting that adolescents are more likely to conform when their parents and peers endorse positive attitudes and resist conformity when they endorse negative ones (Do et al., 2020). In line with previously observed gender differences in prosocial and antisocial behaviours (Burt et al., 2018; for reviews, see Sutter et al., 2019; Van der Graaff et al., 2018), females had lower antisocial and higher prosocial first‐ratings than males. We also found that females were less socially influenced by antisocial ratings than males. While this finding that emerged from the sensitivity analyses was not part of our main hypotheses, it is in line with a school‐based study which, using self‐report and peer nominations of antisocial behaviour, found that girls who were characterised by consistently elevated levels of antisocial behaviour were less affected by deviant peers than boys (Van Lier et al., 2005). These sensitivity analyses highlight the importance of considering gender when investigating prosocial and antisocial decision‐making during adolescence.

To investigate the impact of mindfulness training in reducing antisocial and prosocial influence, the current study compared the effectiveness of mindfulness training and a control training programme in a sample of adolescents. We hypothesised that MT would have had an impact on susceptibility to both types of social influence (hypothesis 2). We found that participants were less influenced by antisocial, but not prosocial, ratings following mindfulness training (hypothesis 2a). However, this was also true for SST training, and therefore the effect of MT was not significantly different from the active control training (hypothesis 2b).

We expected mindfulness training to have a greater impact on social influence than an active control training as it has been previously correlated with executive processes, especially self‐control (Elkins‐Brown et al., 2017; Masicampo & Baumeister, 2007). However, contrary to literature on the role of self‐control in decreasing susceptibility to social influence (Meldrum et al., 2013), our results suggest that mindfulness training might not impact social influence through a mechanism that is distinct to other types of socioemotional training. In fact, exploratory analyses also suggested that there were no differences between interventions in executive functioning measures related to self‐control, such as emotional control and inhibition. Therefore, it is possible that the observed decrease in influence in the antisocial condition is driven by common elements in both training programmes. For example, both training programmes focus on building self‐esteem and cultivating kindness and gratitude, which are associated with less antisocial behaviour (Bono et al., 2019; Donnellan et al., 2005; Gao et al., 2020). It is possible that cultivating kindness, gratitude and increasing self‐esteem as a result of undergoing one or the other of the programmes led to a reduction in social influence in the antisocial condition.

Given that a passive no‐training control group was not included in this study, we cannot exclude possibilities of potential confounds. One possible confound is age, and previous research on social influence suggests that susceptibility to both prosocial and antisocial influence decreases with age across adolescence (Foulkes et al., 2018; Knoll et al., 2015, 2017). Therefore, the reduction in influence in the antisocial condition observed pre‐ and post‐training could be due to the increase in age over the weeks between the two testing sessions. However, this seems unlikely for two reasons. First, there is no evidence that age‐related changes in social influence can occur over such a short time‐period (9–10 weeks). Second, there is no evidence from other studies, or in our baseline data (Ahmed et al., 2020), that increasing age is associated with a change in antisocial influence more than prosocial influence. A second explanation for the reduction of influence in antisocial ratings pre‐ to post‐training is an effect of practise as participants become more familiar with the task. To our knowledge, no previous studies have investigated changes in susceptibility to social influence over a short period (in any age group). In addition, as above, this would not explain the asymmetry in the results as practise or carry‐over effects might be expected to affect prosocial as well as antisocial influence.

## 5 CONCLUSION

The present study compared the effect of mindfulness training with a control student skills training programme on adolescents' susceptibility to prosocial and antisocial influence in adolescence. We found that participants were less influenced by antisocial, but not prosocial, ratings following both training programmes. This result highlights the importance of considering the type of social behaviours (whether behaviours are prosocial or antisocial) when understanding social influence during adolescence. However, it is important to note that the basis of this change cannot be discerned without including a no‐training control group. Future studies should investigate whether and how social–emotional training interventions might be effective at reducing susceptibility to antisocial influence in adolescence.

## AUTHOR CONTRIBUTIONS

**Jovita Tung Leung:** Data curation; formal analysis; investigation; methodology; project administration; resources; visualization; writing – original draft; writing – review and editing. **Blanca Piera Pi‐Sunyer:** Data curation; formal analysis; investigation; methodology; project administration; visualization; writing – original draft; writing – review and editing. **Saz Ahmed:** Data curation; formal analysis; investigation; methodology; project administration; visualization; writing – original draft; writing – review and editing. **Lucy Foulkes:** Conceptualization; data curation; investigation; methodology; project administration; resources; visualization; writing – review and editing. **Cait Griffin:** Data curation; investigation; methodology; project administration; resources; visualization; writing – review and editing. **Ashok Sakhardande:** Data curation; investigation; methodology; project administration; resources; visualization; writing – review and editing. **Marc Bennett:** Investigation; project administration; writing – review and editing. **Darren Dunning:** Conceptualization; data curation; investigation; methodology; project administration; resources; software; writing – review and editing. **Kirsty Griffiths:** Data curation; investigation; methodology; project administration; resources; software; writing – review and editing. **Jenna Parker:** Data curation; investigation; methodology; project administration; resources; software; writing – review and editing. **Willem Kuyken:** Conceptualization; funding acquisition; methodology; writing – review and editing. **Mark Williams:** Conceptualization; funding acquisition; methodology; supervision; writing – review and editing. **Tim Dalgleish:** Conceptualization; data curation; funding acquisition; investigation; methodology; project administration; supervision; writing – review and editing. **Sarah‐Jayne Blakemore:** Conceptualization; data curation; formal analysis; funding acquisition; investigation; methodology; project administration; supervision; writing – original draft; writing – review and editing.

### PEER REVIEW

The peer review history for this article is available at https://publons.com/publon/10.1002/icd.2386.

## Supporting information


**Figure S1.** Effect of Age and Social Condition on First Ratings.
**Figure S2.** Effect of Gender and Social Condition on First Ratings.
**Figure S3.** Effect of Gender and Social Condition on Social Influence.
**Figure S4.** Effect of Age on Social Influence.
**Figure S5.** Effect of IQ on Social Influence.
**Table S1.** Full list of social influence task scenarios.
**Table S2.** Model 1 Output – First Ratings.
**Table S3.** Model 1 and CM Estimates – First Ratings.
**Table S4.** Model 2 Output – Change in Rating.
**Table S5.** Interaction between Delta Rating, Social Condition and Direction of Influence (Model 2) – Contrast Estimates.
**Table S6.** Interaction between Delta Rating, Social Condition and Testing Session (Model 2) – Contrast Estimates.
**Table S7.** Model 2 and CM Estimates – Change in Rating.
**Appendix S1.** Sensitivity analyses.

## Data Availability

The fully anonymised data that support the findings of this study are available from the corresponding author upon reasonable request.

## References

[icd2386-bib-0001] Ahmed, S. , Foulkes, L. , Leung, J. T. , Griffin, C. , Sakhardande, A. L. , Bennett, M. , Dunning, D. L. , Griffiths, K. , Parker, J. , Kuyken, W. , Williams, J. M. G. , Dalgleish, T. , & Blakemore, S.‐J. (2020). Susceptibility to prosocial and antisocial influence in adolescence. Journal of Adolescence, 84, 56–68. 32858504 10.1016/j.adolescence.2020.07.012 PMC7674583

[icd2386-bib-0003] Atlantic Education Consultants . (2013). Student success skills. https://studentsuccessskills.com/

[icd2386-bib-0004] Barr, D. J. , Levy, R. , Scheepers, C. , & Tily, H. J. (2013). Random effects structure for confirmatory hypothesis testing: Keep it maximal. Journal of Memory and Language, 68(3), 255–278. 10.1016/j.jml.2012.11.001 PMC3881361 24403724

[icd2386-bib-0005] Bates, D. , Mächler, M. , Bolker, B. , & Walker, S. (2015). Fitting linear mixed‐effects models using lme4. Journal of Statistical Software, 67(1), 1‐48. 10.18637/jss.v067.i01

[icd2386-bib-0006] Bishop, S. R. , Lau, M. , Shapiro, S. , Carlson, L. , Anderson, N. D. , Carmody, J. , … Devins, G. (2004). Mindfulness: A proposed operational definition. Clinical Psychology: Science and Practice, 11(3), 230–241.

[icd2386-bib-0007] Bögels, S. , Hoogstad, B. , van Dun, L. , de Schutter, S. , & Restifo, K. (2008). Mindfulness training for adolescents with externalizing disorders and their parents. Behavioural and Cognitive Psychotherapy, 36(2), 193–209. 10.1017/S1352465808004190

[icd2386-bib-0008] Bono, G. , Froh, J. J. , Disabato, D. , Blalock, D. , McKnight, P. , & Bausert, S. (2019). Gratitude's role in adolescent antisocial and prosocial behavior: A 4‐year longitudinal investigation. The Journal of Positive Psychology, 14(2), 230–243.

[icd2386-bib-0010] Burt, S. A. , Slawinski, B. L. , & Klump, K. L. (2018). Are there sex differences in the etiology of youth antisocial behavior? Journal of Abnormal Psychology, 127(1), 66–78. 10.1037/abn0000324 29369668 PMC5788285

[icd2386-bib-0011] Cheang, R. , Gillions, A. , & Sparkes, E. (2019). Do mindfulness‐based interventions increase empathy and compassion in children and adolescents: A systematic review. Journal of Child and Family Studies, 28(7), 1765–1779. 10.1007/s10826-019-01413-9

[icd2386-bib-0012] Chierchia, G. , Piera Pi‐Sunyer, B. , & Blakemore, S.‐J. (2020). Prosocial influence and opportunistic conformity in adolescents and young adults. Psychological Science, 31(12), 1585–1601. 10.1177/0956797620957625 33226891 PMC7734552

[icd2386-bib-0013] Choukas‐Bradley, S. , Giletta, M. , Cohen, G. L. , & Prinstein, M. J. (2015). Peer influence, peer status, and prosocial behavior: An experimental investigation of peer socialization of adolescents' intentions to volunteer. Journal of Youth and Adolescence, 44(12), 2197–2210. 10.1007/s10964-015-0373-2 26525387 PMC5985442

[icd2386-bib-0050] Department of Health and Department for Education . (2017). Transforming Children and Young People's Mental Health Provision: A Green Paper. https://assets.publishing.service.gov.uk/government/uploads/system/uploads/attachment_data/file/664855/Transforming_children_and_young_people_s_mental_health_provision.pdf

[icd2386-bib-0014] Do, K. T. , McCormick, E. M. , & Telzer, E. H. (2020). Neural sensitivity to conflicting attitudes supports greater conformity toward positive over negative influence in early adolescence. Developmental Cognitive Neuroscience, 11, 100837. 10.1016/j.dcn.2020.100837 PMC7562935 32830094

[icd2386-bib-0015] Donald, J. N. , Sahdra, B. K. , Zanden, B. V. , Duineveld, J. J. , Atkins, P. W. B. , Marshall, S. L. , & Ciarrochi, J. (2019). Does your mindfulness benefit others? A systematic review and meta‐analysis of the link between mindfulness and prosocial behaviour. British Journal of Psychology, 110(1), 101–125. 10.1111/bjop.12338 30094812

[icd2386-bib-0016] Donnellan, M. B. , Trzesniewski, K. H. , Robins, R. W. , Moffitt, T. E. , & Caspi, A. (2005). Low self‐esteem is related to aggression, antisocial behavior, and delinquency. Psychological Science, 16(4), 328–335. 15828981 10.1111/j.0956-7976.2005.01535.x

[icd2386-bib-0017] Dunning, D. L. , Griffiths, K. , Kuyken, W. , Crane, C. , Foulkes, L. , Parker, J. , & Dalgleish, T. (2019). Research review: The effects of mindfulness‐based interventions on cognition and mental health in children and adolescents–a meta‐analysis of randomized controlled trials. Journal of Child Psychology and Psychiatry, 60(3), 244–258. 30345511 10.1111/jcpp.12980 PMC6546608

[icd2386-bib-0051] Elkins‐Brown, N. , Teper, R. , & Inzlicht, M. (2017). How mindfulness enhances self‐control. In Mindfulness in social psychology (pp. 65–78). Routledge.

[icd2386-bib-0019] Foulkes, L. , Leung, J. T. , Fuhrmann, D. , Knoll, L. J. , & Blakemore, S.‐J. (2018). Age differences in the prosocial influence effect. Developmental Science, 21, e12666. 10.1111/desc.12666 29658168 PMC6221149

[icd2386-bib-0020] Franco, C. , Amutio, A. , López‐González, L. , Oriol, X. , & Martínez‐Taboada, C. (2016). Effect of a mindfulness training program on the impulsivity and aggression levels of adolescents with behavioral problems in the classroom. Frontiers in Psychology, 7, 1385. 10.3389/fpsyg.2016.01385 27713709 PMC5031764

[icd2386-bib-0021] Gao, F. , Yao, Y. , Yao, C. , Xiong, Y. , Ma, H. , & Liu, H. (2020). Moderating effect of eamily support on the mediated relation between negative life events and antisocial behavior tendencies via self‐esteem among chinese adolescents. Frontiers in Psychology, 11, 1769. 32903752 10.3389/fpsyg.2020.01769 PMC7438802

[icd2386-bib-0024] Kabat‐Zinn, J. (1990). Full catastrophe living: How to cope with stress, pain and illness using mindfulness meditation. Piatkus.

[icd2386-bib-0025] Knoll, L. J. , Leung, J. T. , Foulkes, L. , & Blakemore, S.‐J. (2017). Age‐related differences in social influence on risk perception depend on the direction of influence. Journal of Adolescence, 60, 53–63. 10.1016/j.adolescence.2017.07.002 28753485 PMC5614112

[icd2386-bib-0026] Knoll, L. J. , Magis‐Weinberg, L. , Speekenbrink, M. , & Blakemore, S.‐J. (2015). Social influence on risk perception during adolescence. Psychological Science, 26(5), 583–592. 10.1177/0956797615569578 25810453 PMC4426139

[icd2386-bib-0027] Lenth, R. , Singmann, H. , Love, J. , Buerkner, P. , & Herve, M. (2018). Emmeans: Estimated marginal means, aka least‐squares means (R package version 1.3.0) [computer software].

[icd2386-bib-0052] Marshal, M. P. , Molina, B. S. , & Pelham, W. E., Jr. (2003). Childhood ADHD and adolescent substance use: an examination of deviant peer group affiliation as a risk factor. Psychology of Addictive Behaviors, 17(4), 293. 14640825 10.1037/0893-164X.17.4.293 PMC3652274

[icd2386-bib-0028] Masicampo, E. J. , & Baumeister, R. F. (2007). Relating mindfulness and self‐regulatory processes. Psychological Inquiry, 18(4), 255–258.

[icd2386-bib-0029] Meldrum, R. C. , Miller, H. V. , & Flexon, J. L. (2013). Susceptibility to peer influence, self‐control, and delinquency. Sociological Inquiry, 83(1), 106–129.

[icd2386-bib-0030] Mindfulness in Schools Project . (2009) .b Curriculum (Age 11–18). https://studentsuccessskills.com/

[icd2386-bib-0031] Monahan, K. C. , Steinberg, L. , & Cauffman, E. (2009). Affiliation with antisocial peers, susceptibility to peer influence, and antisocial behavior during the transition to adulthood. Developmental Psychology, 45(6), 1520–1530. 10.1037/a0017417 19899911 PMC2886974

[icd2386-bib-0053] Moutoussis, M. , Dolan, R. J. , & Dayan, P. (2016). How people use social information to find out what to want in the paradigmatic case of inter‐temporal preferences. PLoS Computational Biology, 12(7), e1004965. 27447491 10.1371/journal.pcbi.1004965 PMC4957786

[icd2386-bib-0032] Oberle, E. , Schonert‐Reichl, K. A. , Lawlor, M. S. , & Thomson, K. C. (2012). Mindfulness and inhibitory control in early adolescence. The Journal of Early Adolescence, 32(4), 565–588.

[icd2386-bib-0033] R Core Team . (2013). R: A language and environment for statistical computing. R Foundation for Statistical Computing.

[icd2386-bib-0034] Riggs, N. R. , Black, D. S. , & Ritt‐Olson, A. (2015). Associations between dispositional mindfulness and executive function in early adolescence. Journal of Child and Family Studies, 24, 2745–2751. 10.1007/s10826-014-0077-3

[icd2386-bib-0035] Sawyer, S. M. , Azzopardi, P. S. , Wickremarathne, D. , & Patton, G. C. (2018). The age of adolescence. The Lancet Child & Adolescent Health, 2(3), 223–228. 30169257 10.1016/S2352-4642(18)30022-1

[icd2386-bib-0036] Segal, Z. V. , Teasdale, J. D. , Williams, J. M. , & Gemar, M. C. (2002). The mindfulness‐based cognitive therapy adherence scale: Inter‐rater reliability, adherence to protocol and treatment distinctiveness. Clinical Psychology & Psychotherapy, 9(2), 131–138.

[icd2386-bib-0037] Sijtsema, J. J. , & Lindenberg, S. M. (2018). Peer influence in the development of adolescent antisocial behavior: Advances from dynamic social network studies. Developmental Review, 50, 140–154. 10.1016/j.dr.2018.08.002

[icd2386-bib-0038] Sutter, M. , Zoller, C. , & Glätzle‐Rützler, D. (2019). Economic behavior of children and adolescents–a first survey of experimental economics results. European Economic Review, 111, 98–121.

[icd2386-bib-0054] Taylor, C. (2018). The reliability of free school meal eligibility as a measure of socio‐economic disadvantage: Evidence from the millennium cohort study in Wales. British Journal of Educational Studies, 66(1), 29–51.

[icd2386-bib-0040] Van der Graaff, J. , Carlo, G. , Crocetti, E. , Koot, H. M. , & Branje, S. (2018). Prosocial behavior in adolescence: Gender differences in development and links with empathy. Journal of Youth and Adolescence, 47(5), 1086–1099. 29185207 10.1007/s10964-017-0786-1 PMC5878203

[icd2386-bib-0041] Van Hoorn, J. , van Dijk, E. , Meuwese, R. , Rieffe, C. , & Crone, E. A. (2016). Peer influence on prosocial behavior in adolescence. Journal of Research on Adolescence, 26(1), 90–100. 10.1111/jora.12173

[icd2386-bib-0042] Van Lier, P. A. , Vitaro, F. , Wanner, B. , Vuijk, P. , & Crijnen, A. A. (2005). Gender differences in developmental links among antisocial behavior, friends' antisocial behavior, and peer rejection in childhood: Results from two cultures. Child Development, 76(4), 841–855. 16026500 10.1111/j.1467-8624.2005.00881.x

