# Supplementary material for: Susceptibility to prosocial and antisocial influence in adolescence following mindfulness training
Source: Infant Child Dev. 2022 Dec 4;32(1):e2386. doi: 10.1002/icd.2386 (PMC10078582; doi:10.1002/icd.2386)
Supplement: Supplementary file 1 — Figure S1. Effect of Age and Social Condition on First Ratings. Figure S2. Effect of Gender and Social Condition on First Ratings. Figure S3. Effect of Gender and Social Condition on Social Influence. Figure S4. Effect of Age on Social Influence. Figure S5. Effect of IQ on Social Influence. Table S1. Full list of social influence task scenarios. Table S2. Model 1 Output – First Ratings. Table S3. Model 1 and CM Estimates – First Ratings. Table S4. Model 2 Output – Change in Rating. Table S5. Interaction between Delta Rating, Social Condition and Direction of Influence (Model 2) – Contrast Estimates. Table S6. Interaction between Delta Rating, Social Condition and Testing Session (Model 2) – Contrast Estimates. Table S7. Model 2 and CM Estimates – Change in Rating. Appendix S1. Sensitivity analyses. [file ICD-32-0-s001.docx]

**Supplemental Material**

**Supplemental Information**

*Supplemental information 1: Sensitivity analyses*

We ran a series of sensitivity analyses, or Control Models (CMs), to account for a number of potentially important factors that could have influenced our findings. We did this by including an additional regressor to the best fitting model for each dependent variable and then using Type III Wald *χ*^2^ tests to test the robustness of our findings. Additionally, one CM used Type III Wald *χ*^2^ tests on the best fitting model excluding a subset of trials containing extreme values in the relevant dependent variable. The CMs for both Model 1 and Model 2 were as follows:

CM1) Age, as well as its interaction with social condition. Age has consistently been found to be a predictor of decreased social influence (Knoll et al., 2017; 2015; Foulkes et al., 2018) and was therefore included to account for potential variance in social influence explained by participants of different ages (fixed effect). We additionally interacted age with social condition, as age differences in antisocial and prosocial behaviour have been previously reported (Ahmed et al., 2020).

CM2) Gender (female = 0) and CM3) IQ, were added based on literature suggesting gender differences in social processing (e.g. Flannery & Smith, 2016), effects of initial preferences on social influence (e.g. Wei et al., 2016) and effects IQ on social cognition (Choudhury, Blakemore & Charman 2006). Gender was additionally interacted with social condition, as gender differences in prosocial and antisocial behaviour have been previously reported (Ahmed et al., 2020).

CM4) Number of homework assignments completed, and CM5) Total attendance were added as covariates as they could reflect participation in the training programmes. Any conclusions about the effectiveness of the interventions should consider these factors.

CM6) Testing group size at pre-training, CM7) Testing group size at post-training, and CM8) Average training group size, were added based on literature suggesting that social influence could be influenced by the mere presence of peers (e.g. van Hoorn et al., 2016 and Gardner & Steinberg, 2005).

CM9) Excluding extreme values, as determined by excluding trials in rating 1 and change in rating with a z-score > 4 (Chierchia et al., 2020). This CM did not converge during Model 2 sensitivity analyses, and thus the random effects structure was progressively simplified, with the final model only including social condition and testing session random slopes to vary by participant.

C10) First Ratings (Model 2 only) was included to account for differences in social influence that could emerge from baseline ratings (Knoll et al., 2015). Given that first ratings varied across social condition differently across age and gender in our Model 1 CM1 and CM2 (see Figure S1 and S2), we interacted these terms with first ratings in the Model 2 CM1 and CM2.

Significant omnibus tests were robust to all control models.

Based on a reviewer’s suggestion, we also ran an exploratory analysis predicting how self-control differed between interventions. We compared nested linear mixed effects models using data collected using the emotional control and inhibition subscales of the Behaviour Rating Inventory of Executive Functioning (BRIEF), which are both relevant to self-control. The results show that a model including the type of intervention as a predictor does not explain additional variance in either inhibition (*χ*^2^(2) = .22; *p* = .896) or emotional control (*χ*^2^(2) = 2.70 *p* = .259). Therefore, mindfulness training did not seem to influence these aspects of self-control measured by the BRIEF differently from the active control training.

*Supplemental information 2: Assessment criteria for rating videotapes*

Instructions. This adherence checklist has each key element for each lesson so the rater can tick off that it was delivered. There are a few things in each lesson which are MUST HAVES, and there are a lot of NICE TO HAVES. ** are placed next to the things that are the MUST HAVES. Everything else is NICE TO HAVES. For example, a teacher should not be evaluated negatively if, say, they don’t use the black-cab example at the start. They might decide this would not connect with their class and leave it out. On the other hand, they must do the ‘hands’ activity, or an equivalent, as this is the first real practice that kids do.

The teacher might begin a lesson with a review of home practice. This can be very effective when enough pupils have done the home practice and the teacher is able to harvest feedback, but it is not a requirement.

**Mindfulness Training Checklist**

*Lesson 1*

- **Clear introduction and rationale
- **Teacher tailors the introduction to the group, in terms of key dimensions such as engagement/motivation, level, size, conscript vs volunteer
- Black Cab example
- Identify neuroscientific link between changes in the brain and amount / frequency of practice ie. brain training
- **Mindfulness of hands practice.
- How the mind and body are inextricably linked. Mind/body connection is taught / illustrated.
- Kung Fu Panda clip is used and facilitates discussion about it
- **brief .b practice. May only be a few seconds long, but if shorter evidence that it builds on earlier mindfulness practices.
- Getting out of a ‘bad place’
- **[Lack of] Mind control exercises – Polar Bear
- Examples of Mindfulness being used which connect with the class.
- **Teacher facilitates *intrinsic motivation* for participating in the course without being prescriptive about outcomes. Emphasis on **possibilities**
- Ground rules are clear
- Clear explanation of ‘Searchlight of attention’ (using torch)
- **Practice 1 ‘Play attention’
- **Puppy explanation
- **Practice 2 – puppy training using a 1- or 2-minute silence
- Finger breathing practice and inquiry
- **Home Practice

*Lesson 2*

- ‘My mind feels, my body feels…’ practice
- Animal minds explanation e.g. monkey, elephant, hippo
- **Discussion of importance of –‘turning towards’ the animal mind with curiosity and kindness.
- **FOFBOC practice and inquiry
- What is the point? Explanation
- **Home practice

*Lesson 3*

- Worry – introduction
- **7/11 practice
- **Two modes of mind
- ‘The mind tells stories...’ explanation
- ‘Sam was on the way to school’ activity and discussion
- **Scenario discussion
- **Overthinking and rumination explanation.
- Home practice discussion
- **Beditation practice and inquiry
- **Home practice

*Lesson 4*

- **Autopilot explanation
- **‘Savouring chocolate’ eating practice and inquiry
- **Anticipation for the ‘Chilli’ eating practice and inquiry
- ‘Tingling of likes and dislikes’ explanation
- **Explanation of gap between stimulus and response
- **‘.b’ practice
- **Home practice

*Lesson 5 (Highly dependent on space available. This checklist is not used in the summative adherence scores and is collected for the record only.)*

- Home practice and recap of practices so far
- **Introduction to moving mindfully
- **Standing practice
- Explanation of ‘flow’ or being ‘in the zone’
- Video clip
- **Mindful walking and inquiry
- **Home practice

*Lesson 6*

- Introduction ‘How is the traffic in your mind’
- Animation clip
- **Mindfulness of sounds and thoughts practice: ‘Stepping Back’ and inquiry
- Links between repetitive thought and neural pathways
- **‘Thought buses’ explanation and activity
- **‘How to stay at the bus stop’ Standing practice
- **Home practice

*Lesson 7*

- Introduction – Difficulties happen to everyone.
- **In what situations do you feel stress?
- ** What happens in your mind, body and actions when you are stressed
- Explanation of stress and its’ effects.
- **Experience of Stress Practice and inquiry
- **Drawing of stress signature
- **Camp-fire practice / or other short ‘settling’ practice
- **Home practice

*Lesson 8*

- **Introduction - mentioning the term ‘heartfulness’
- **Grape eating practice (or other fruit)
- **‘Since the day you were born…’ activity
- Video clip – Alice Hertz-Sommer and/or Soul Pancake clip

**Student Success Training Checklist**

*Lesson 1*

- Introduction to SSS
- Building a caring, supportive and encourage community using the Looks Like, Sounds Like, Feels Like activity
- Explain the maze activity and go through optimism cheer
- Talk through the Imagine slide
- Explain how the term Kaizen is used.
- Using the Austin's Butterfly video, explain to students how Austin has improved his initial drawing
- Look Good/Feeling Good sheet - show examples of goals and plan.

*Lesson 2*

- Recap what they learned in the last session
- Show goose- clip and explain symbolism of V- Shape
- Looking Good/ Feeling Good sheet - rate progress and hear about students’ successes. Remind them how you expect them to listen (Eyes, Ears, Heart)
- Brain Gym - remind students that taking breaks and moving to help boost concentration
- Students to read '5 test taking strategies' and discuss which one they are already familiar with and which ones they use or might like to use in the future
- Revisit optimism cheer and complete matching activity together
- Revisit imagine slide
- Complete seven keys sheet
- To understand the concept of method if loci using the Homer Simpson example. Help students to memorise the food items
- Go through goal setting exercise and for students to practice listening skills and develop empathy
- Show pyramid slide
- Kaizen activity

*Lesson 3*

- Recap what they learned in the last session
- Go through Looking Good/ Feeling Good sheet
- Goal reporting, progress monitoring, success sharing and goal setting
- Show clip from Disney's Mulan
- Revisit optimism cheer
- Looks Like, Sounds Like, Feels Like activity
- Talk through Imagine slide
- Play clip of babies' to music and explain Keep Kool Tunes
- Brain gym
- Boosting memory (slide 18) activity
- Go through seven keys sheet
- Goal setting
- Show pyramid slide
- Revisit Kaizen task

*Lesson 4*

- Recap what they learned in the last session
- Go through Looking Good/ Feeling Good sheet
- Goal reporting, progress monitoring, success sharing and goal setting
- Revisit optimism cheer
- Talk through Imagine slide
- Brain gym
- Explain story outlines
- Play Toy Story clip and discuss the 'Middle' of the story
- Encouraging things to say and do activity
- Go through seven keys sheet
- Looks Like, Sounds Like, Feels Like activity
- Show pyramid slide
- Revisit Kaizen task
- Goal setting

*Lesson 5*

- Recap what they learned in the last session
- Go through Looking Good/ Feeling Good sheet
- Goal reporting, progress monitoring, success sharing and goal setting
- Revisit optimism cheer
- Talk through Imagine slide
- Brain gym
- Story outlines and student story telling activity
- Go through positive self-talk worksheet
- Revisit keep kool tunes
- Go through seven keys sheet
- Looks Like, Sounds Like, Feels Like activity
- Show pyramid slide
- Revisit Kaizen task
- Goal setting
- Preview booster session

*Lesson 6*

- Recap what they learned in the last session
- Go through Looking Good/ Feeling Good sheet
- Goal reporting, progress monitoring, success sharing and goal setting
- Revisit optimism cheer
- Talk through Imagine slide
- Brain gym
- Show video of animals collaborating and discuss with students
- Revisit keep kool tunes
- Go through seven keys sheet
- Looks Like, Sounds Like, Feels Like activity
- Show pyramid slide
- Revisit Kaizen task
- Goal setting

*Lesson 7*

- Recap what they learned in the last session
- Go through Looking Good/ Feeling Good sheet
- Goal reporting, progress monitoring, success sharing and goal setting
- Revisit optimism cheer
- Talk through Imagine slide
- Brain gym
- Story outlines and student story telling activity
- Positive self-talk worksheet
- Revisit keep kool tunes
- Go through seven keys sheet
- Looks Like, Sounds Like, Feels Like activity
- Show pyramid slide
- Revisit Kaizen task
- Goal setting
- Preview booster session

*Lesson 8*

- Recap what they learned in the last session
- Go through Looking Good/ Feeling Good sheet
- Goal reporting, progress monitoring, success sharing and goal setting
- Revisit optimism cheer
- Talk through Imagine slide
- Brain gym
- Discuss strategies to manage test anxiety
- Go through seven keys sheet
- Looks Like, Sounds Like, Feels Like activity
- Show pyramid slide
- Revisit Kaizen task
- Goal setting

*Supplemental information 3: Nested model comparisons and model syntaxes*

We ran a series of nested model comparisons to find the best fitting model to predict variance in *first ratings* (Model 1) and *change in ratings* (Model 2). We progressively included predictors to a null model for each dependent variable to investigate whether these would improve model fit.

***Model 1: First Ratings.*** We included the predictors in the following order: social condition, testing session and type of training. We found that including social condition predicted first ratings better than a null model not including this term (Δ*χ*^2^(3) = 3580.1, *p* < .001). Interacting social condition with testing session did not improve the model fit (Δ*χ*^2^(2) = 1.79, *p* = .408), and neither did including testing session as a main effect (Δ*χ*^2^(1) = .94, *p* = .332). This was also the case when interacting social condition with the type of intervention (Δ*χ*^2^(2) = 1.09, *p* = .580), as well as including this term as a main effect (Δ*χ*^2^(1) = .67, *p* = .413). Therefore, the best model is one that predicts first ratings from social condition only. Random effects of social condition were clustered by participant as this was the only (within-subjects) predictor included as a fixed effect.

The equation for the best fitting Model 1 is as follows:

$${First Rating}_{i}$$

$$=Intercept+ {Random Intercept}_{i}$$

$$+\left( \beta_{1}+ {Random Slope}_{1i} \right)X {Social Condition}_{i}+{error}_{i}$$

The R syntax for Model 1 is as follows:

$$First Rating \sim Social Condition+\left( Social Condition \right| Participant)$$

***Model 2: Change in Ratings.*** We included the predictors in the following order: delta rating, social condition, direction of influence, testing session and type of intervention. Delta rating was included as the first and main predictor of Model 2, as we operationalised social influence as the effect of delta rating on change in rating (see Foulkes et al., 2018; Knoll et al., 2017; 2015). Therefore, all variables were interacted with change in rating, as we were primarily interested on the effect of all predictors on social influence. We found that delta rating predicted change in rating better than a null model (Δ*χ*^2^(1) = 1586.1, *p* < .001). Interacting social condition with the delta rating fit better than a model without this interaction (Δ*χ*^2^(4) = 497.52, *p* < .001). In addition, interacting the delta rating and social condition with the direction of influence improved model fit (Δ*χ*^2^(7) = 848.45, *p* < .001), as well as further interacting these terms with testing session (Δ*χ*^2^(23) = 158.07, *p* < .001). Further, interacting delta rating, social condition, direction of influence and testing session with the type of intervention did not improve model fit (Δ*χ*^2^(16) = 22.61, *p* = .125), and neither did a model adding the lower-level interaction of type of intervention and delta rating (Δ*χ*^2^(2) = 4.10, *p* = .129). For this reason, *type of intervention* was not included as a predictor in Model 2. Finally, a simpler model including the three-way interaction of delta rating, social condition, and direction of influence, as well as the three-way interaction of delta rating, social condition and testing session improved model fit compared to a model including the four-way interaction between these terms (Δ*χ*^2^(4) = 3.20, *p* = .525), and therefore the simpler model was chosen as the best fitting model for change in rating. The maximal random slopes of the within-subject factors included in the final model (i.e. social condition, testing session and direction of influence; Barr et al., 2013) were the interactions between social condition and testing session, and the interaction between direction of influence and testing session, both clustered by participant.

The equation for the best fitting Model 2 is as follows:

$${Change in Rating}_{i}$$

$$=Intercept+ {Random Intercept}_{i}+\left( \beta_{1} \right) X {Delta Rating}_{i}$$

$$+\left( \beta_{2}+{Random Slope}_{2i} \right) X {Social Condition}_{i}$$

$$+\left( \beta_{3}+{Random Slope}_{3i} \right)X {Direction of Influence}_{i}$$

$$+\left( \beta_{4}+{Random Slope}_{4i} \right)X {Testing Session}_{i}$$

$$+\left( \beta_{5} \right) X {Delta Rating}_{i} X {Social Condition}_{i}$$

$$+\left( \beta_{6} \right) X {Delta Rating}_{i} X {Direction of Influence}_{i}$$

$$+\left( \beta_{7} \right) X {Social Condition}_{i} X {Direction of Influence}_{i}$$

$$+\left( \beta_{8} \right) X {Delta Rating}_{i} X {Testing Session}_{i}$$

$$+\left( \beta_{9}+{Random Slope}_{9i} \right) X S{ocial Condition}_{i} X {Testing Session}_{i}$$

$$+\left( {Random Slope}_{10i} \right) X {Direction of Influence}_{i} X T{esting Session}_{i}$$

$$+\left( \beta_{11} \right) X {Delta Rating}_{i} X {Social Conditon}_{i} X {Direction of Influence}_{i}$$

$$+\left( \beta_{12} \right) X {Delta Rating}_{i} X S{ocial Condition}_{i} X {Testing Session}_{i}+{error}_{i}$$

The R syntax for Model 2 is as follows:

$$First Rating \sim$$

$$Delta Rating X Social Condition X Direction of Influence$$

$$+ Delta Rating X Social Condition X Testing Session$$

$$+ \left( \begin{aligned} Social Condition X Testing Session \\ +Direction of Influence X Testing Session \end{aligned} \right| Participant)$$

**Supplemental Tables**

*Table S1. Full list of social influence task scenarios*

| **Prosocial scenarios** | **Antisocial scenarios** |
| --- | --- |
| Visit a friend when they are ill | Shout at a family member in an argument |
| Care for a friend when they are ill | Shout at a friend in an argument |
| Give up your seat for a friend on the bus | Take something that doesn’t belong to you |
| Give up your seat for a family member on the bus | Laugh at a friend when they make a mistake |
| Give up your seat to a stranger on the bus | Laugh at a classmate when they make a mistake |
| Give up your seat for a friend on the train | Laugh at a family member when they make a mistake |
| Give up your seat to a stranger on the train | Look through a friend's phone without asking |
| Carry a friend's bag for them | Look through a classmate's phone without asking |
| Carry a family member's bag for them | Look through a family member's phone without asking |
| Make a friend a birthday card | Push in front of friend in a queue |
| Make a family member a birthday card | Gossip about a classmate |
| Buy a friend a birthday card | Gossip about a friend |
| Buy a family member a birthday card | Gossip about a friend online |
| Stand up for a classmate when they are being teased | Talk about a friend behind their back |
| Defend a classmate when they are being bullied | Talk about a classmate behind their back |
| Give something you like to charity | Laugh at someone's clothes |
| Lend a friend your favourite book | Laugh at someone's work |
| Let a friend go ahead of you in a queue | Look through a family member's room when they are away |
| Let a classmate go ahead of you in a queue | Make a mess at home and not clear it up |
| Lend a friend your favourite clothes | Ignore a friend online |
| Raise money for charity | Ignore a friend's Whatsapp messages |
| Buy a friend a birthday present | Make fun of a friend |
| Give money to charity | Make fun of a classmate |
| Show a stranger where to go if they are lost | Tease a friend |
| Make a friend a present | Tease a family member |
| Make a family member a present | Tease a classmate |
| Offer to help around the house | Tell someone's secret |
| Lend a friend money | Trip up a classmate as a joke |
| Lend a family member money | Trip up a friend as a joke |
| Volunteer for a charity | Hit a friend when you are angry |
| Help a stranger if they have fallen | Lie to a teacher to get out of trouble |
| Help a friend with their schoolwork | Lie to a parent to get out of trouble |
| Help a classmate with their schoolwork | Write or draw on a desk at school |
| Share your revision notes with a friend | Answer back to a teacher in a rude way |
| Share your revision notes with a classmate | Pretend you are someone else online |
| Like a friend's post on Facebook | Ignore what a teacher asks you to do |
| Compliment a family member | Blame a friend for something you did wrong |
| Message a friend to see how they are | Blame a classmate for something you did wrong |
| Sponsor a friend for charity | Blame a family member for something you did wrong |
| Sponsor a classmate for charity | Swear at a friend in an argument |
| Sponsor a family member for charity | Swear at a family member in an argument |

*Table S2. Model 1 Output – First Ratings*

|  | **χ^2^** | **Df** | **p-value** |
| --- | --- | --- | --- |
| Intercept | 10302.36 | 1 | <0.001 |
| Social Condition | 572.33 | 1 | <0.001 |

*Table S3. Model 1 and CM Estimates – First Ratings*

|  | Main Model | **CM1)** Main Model + Age | **CM2)** Main Model + Gender | **CM3)** Main Model + IQ | **CM4)** Main Model + Attendance | **CM5)** Main Model + Homework | **CM6)** Main Model + Group Size at T1 | **CM7)** Main Model + Group Size at T2 | **CM8)** Main Model + Average Training Size | **CM9)** Main Model + Outliers |
| --- | --- | --- | --- | --- | --- | --- | --- | --- | --- | --- |
| Intercept | 6.28***  (0.06) | 7.64***  (0.62) | 6.40***  (0.08) | 6.64***  (0.26) | 6.30***  (0.13) | 6.30***  (0.1) | 6.67***  (0.24) | 6.51***  (0.17) | 6.16***  (0.14) | 6.28***  (0.06) |
| Social Condition (Antisocial) | -2.65***  (0.11) | -7.87***  (1.09) | -2.87***  (0.13) | -2.66***  (0.11) | -2.65***  (0.11) | -2.65***  (0.11) | -2.65***  (0.11) | -2.80***  (0.12) | -2.64***  (0.11) | -2.65***  (0.11) |
| Age |  | -0.1*  (0.04) |  |  |  |  |  |  |  |  |
| Age x Social Condition (Antisocial) |  | 0.38***  (0.08) |  |  |  |  |  |  |  |  |
| Gender (Male) |  |  | -0.35**  (0.13) |  |  |  |  |  |  |  |
| Gender (Male) + Social Condition (Antisocial) |  |  | 0.66**  (0.23) |  |  |  |  |  |  |  |
| IQ |  |  |  | 0  (0) |  |  |  |  |  |  |
| Attendance |  |  |  |  | 0  (0.02) |  |  |  |  |  |
| Homework |  |  |  |  |  | -0.01  (0.02) |  |  |  |  |
| Group Size at T1 |  |  |  |  |  |  | -0.03·  (0.02) |  |  |  |
| Group Size at T2 |  |  |  |  |  |  |  | -0.01  (0.01) |  |  |
| Average Training Size |  |  |  |  |  |  |  |  | 0.01  (0.01) |  |

*Table S4. Model 2 Output – Change in Rating*

|  | **χ^2^** | **Df** | **p-value** |
| --- | --- | --- | --- |
| Intercept | 36.17 | 1 | <0.001 |
| Delta Rating | 122.64 | 1 | <0.001 |
| Social Condition | 15.12 | 1 | <0.001 |
| Direction of Influence | 33.00 | 1 | <0.001 |
| Testing Session | 0.08 | 1 | 0.777 |
| Delta Rating X Social Condition | 11.84 | 1 | <0.001 |
| Delta Rating X Direction of Influence | 17.48 | 1 | <0.001 |
| Social Condition X Direction of Influence | 2.79 | 1 | 0.095 |
| Delta Rating X Testing Session | 1.36 | 1 | 0.243 |
| Social Condition X Testing Session | 0.03 | 1 | 0.865 |
| Delta Rating X Social Condition X Direction of Influence | 36.34 | 1 | <0.001 |
| Delta Rating X Social Condition X Testing Session | 6.57 | 1 | 0.010 |

*Table S5. Interaction between Delta Rating, Social Condition and Direction of Influence (Model 2) – Contrast Estimates*

| **Social Condition** | **Direction of Influence** | **Delta Rating Estimate** | **SE** | **p-value** |
| --- | --- | --- | --- | --- |
| Prosocial | *Higher* | 0.15 | 0.01 | <0.001 |
| Prosocial | *Lower* | 0.08 | 0.01 | <0.001 |
| Antisocial | *Higher* | 0.07 | 0.01 | <0.001 |
| Antisocial | *Lower* | 0.15 | 0.01 | <0.001 |

*Table S6. Interaction between Delta Rating, Social Condition and Testing Session (Model 2) – Contrast Estimates*

| **Social Condition** | **Testing Session** | **Delta Rating Estimate** | **SE** | **p-value** |
| --- | --- | --- | --- | --- |
| Prosocial | *Pre-Training* | 0.12 | 0.01 | <0.001 |
| Prosocial | *Post-Training* | 0.11 | 0.01 | <0.001 |
| Antisocial | *Pre-Training* | 0.13 | 0.01 | <0.001 |
| Antisocial | *Post-Training* | 0.09 | 0.01 | <0.001 |

*Table S7. Model 2 and CM Estimates – Change in Rating*

|  | Main Model | **CM1)** Main Model + Age | **CM2)** Main Model + Gender | **CM3)** Main Model + IQ | **CM4)** Main Model + Attendance | **CM5)** Main Model + Homework | **CM6)** Main Model + Group Size at T1 | **CM7)** Main Model + Group Size at T2 | **CM8)** Main Model + Average Training Size | **CM9)** Main Model + Outliers | **CM10)** Main Model + First Rating |
| --- | --- | --- | --- | --- | --- | --- | --- | --- | --- | --- | --- |
| Intercept | 0.33***  (0.05) | 0.33***  (0.05) | 0.33***  (0.05) | 0.32***  (0.05) | 0.33***  (0.05) | 0.33***  (0.05) | 0.33***  (0.05) | 0.35***  (0.06) | 0.33***  (0.05) | 0.40***  (0.04) | 0.32***  (0.06) |
| Delta Rating | 0.15***  (0.01) | 0.25***  (0.05) | 0.15***  (0.01) | 0.28***  (0.03) | 0.17***  (0.02) | 0.17***  (0.02) | 0.16***  (0.03) | 0.17***  (0.02) | 0.15***  (0.02) | 0.08***  (0.01) | 0.15***  (0.01) |
| Social Condition (Antisocial) | -0.26***  (0.07) | -0.26***  (0.07) | -0.26***  (0.07) | -0.26***  (0.07) | -0.26***  (0.07) | -0.26***  (0.07) | -0.26***  (0.07) | -0.31***  (0.07) | -0.26***  (0.07) | -0.30***  (0.05) | -0.26***  (0.07) |
| Direction of Influence (Lower) | -0.40***  (0.07) | -0.41***  (0.07) | -0.40***  (0.07) | -0.41***  (0.07) | -0.40***  (0.07) | -0.40***  (0.07) | -0.40***  (0.07) | -0.41***  (0.07) | -0.40***  (0.07) | -0.52***  (0.06) | -0.40***  (0.07) |
| Testing Session (Post-Training) | 0.01  (0.04) | 0.01  (0.04) | 0.01  (0.04) | 0.01  (0.04) | 0.01  (0.04) | 0.01  (0.04) | 0.01  (0.04) | -0.01  (0.04) | 0.01  (0.04) | 0.01  (0.03) | 0.01  (0.04) |
| Delta Rating x Social Condition (Antisocial) | -0.06***  (0.02) | -0.06  (0.06) | -0.06***  (0.02) | -0.05**  (0.02) | -0.06***  (0.02) | -0.06***  (0.02) | -0.06***  (0.02) | -0.06**  (0.02) | -0.06***  (0.02) | -0.01  (0.01) | -0.06***  (0.02) |
| Delta Rating x Direction of Influence (Lower) | -0.07***  (0.02) | -0.07***  (0.02) | -0.07***  (0.02) | -0.07***  (0.02) | -0.07***  (0.02) | -0.06***  (0.02) | -0.07***  (0.02) | -0.06***  (0.02) | -0.07***  (0.02) | -0.02  (0.01) | -0.08***  (0.02) |
| Social Condition (Antisocial) x Direction of Influence (Lower) | -0.14·  (0.08) | -0.13  (0.08) | -0.14·  (0.08) | -0.13  (0.08) | -0.14·  (0.08) | -0.14·  (0.08) | -0.14·  (0.08) | -0.09  (0.09) | -0.14·  (0.08) | -0.08  (0.07) | -0.14·  (0.08) |
| Delta Rating x Testing Session (Post-Training) | -0.01  (0.01) | -0.01  (0.01) | -0.01  (0.01) | -0.01  (0.01) | -0.01  (0.01) | -0.01  (0.01) | -0.01  (0.01) | -0.02  (0.01) | -0.01  (0.01) | -0.01  (0.01) | -0.01  (0.01) |
| Testing Session (Post-Training) x Social Condition (Antisocial) | -0.01  (0.06) | -0.01  (0.06) | -0.01  (0.06) | -0.02  (0.06) | -0.01  (0.06) | -0.01  (0.06) | -0.01·  (0.06) | 0.03  (0.06) | -0.01  (0.06) | -0.02  (0.04) | -0.01  (0.06) |
| Delta Rating x Social Condition (Antisocial) x Direction of Influence (Lower) | 0.14***  (0.02) | 0.14***  (0.02) | 0.14***  (0.02) | 0.13***  (0.02) | 0.14***  (0.02) | 0.14***  (0.02) | 0.14***  (0.02) | 0.15***  (0.02) | 0.14***  (0.02) | 0.05**  (0.02) | 0.14***  (0.02) |
| Delta Rating x Testing Session (Post-Training) x Social Condition (Antisocial) | -0.03*  (0.01) | -0.03*  (0.01) | -0.03**  (0.01) | -0.03**  (0.01) | -0.03*  (0.01) | -0.03*  (0.01) | -0.03*  (0.01) | -0.03*  (0.01) | -0.03*  (0.01) | -0.02*  (0.01) | -0.03*  (0.01) |
| Delta Rating x Age |  | -0.01*  (0) |  |  |  |  |  |  |  |  |  |
| Delta Rating x Age x Social Condition (Antisocial) |  | 0  (0) |  |  |  |  |  |  |  |  |  |
| Delta Rating x Gender (Male) |  |  | 0  (0.01) |  |  |  |  |  |  |  |  |
| Delta Rating x Gender (Male) x Social Condition (Antisocial) |  |  | 0  (0.01) |  |  |  |  |  |  |  |  |
| Delta Rating x IQ |  |  |  | -0.003***  (0) |  |  |  |  |  |  |  |
| Delta Rating x Attendance |  |  |  |  | 0  (0) |  |  |  |  |  |  |
| Delta Rating x Homework |  |  |  |  |  | 0·  (0) |  |  |  |  |  |
| Delta Rating x Group Size at T1 |  |  |  |  |  |  | 0  (0) |  |  |  |  |
| Delta Rating x Group Size at T2 |  |  |  |  |  |  |  | 0  (0) |  |  |  |
| Delta Rating x Average Training Size |  |  |  |  |  |  |  |  | 0  (0) |  |  |
| Delta Rating x First Rating |  |  |  |  |  |  |  |  |  |  | 0  (0) |

**Supplemental Figures**

*
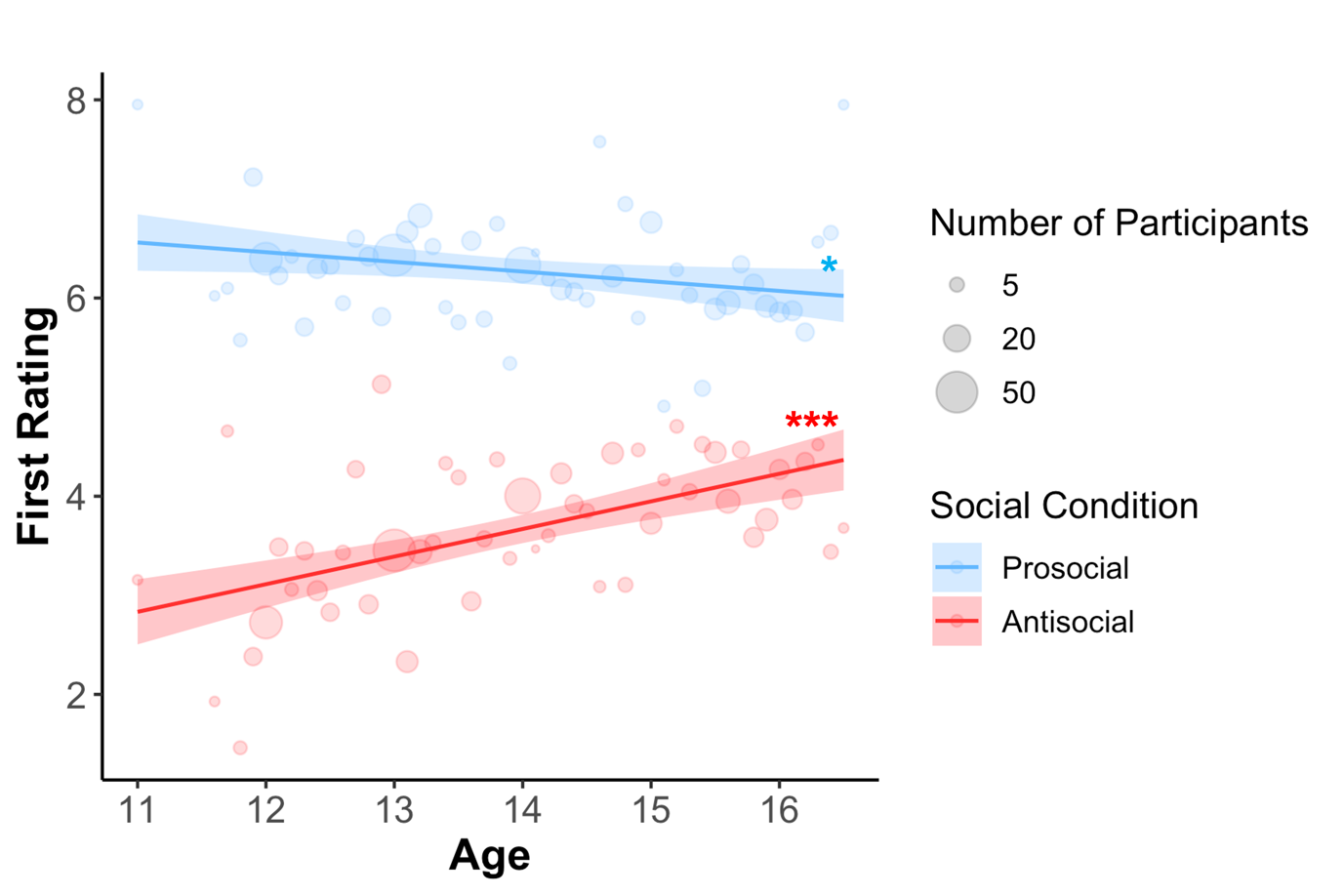
Figure S1. Effect of Age and Social Condition on First Ratings*

The figure shows mean participant-level first ratings grouped by decimal age (bubbles) across prosocial (blue) and antisocial (red) conditions. The lines show model predicted estimates of age on prosocial (blue) as well as antisocial (red) first ratings and corresponding 95% confidence intervals (shaded area). Antisocial first ratings increase significantly with age, while prosocial ratings decrease significantly with age. Asterisks indicate **p* < .05 ****p* < .001


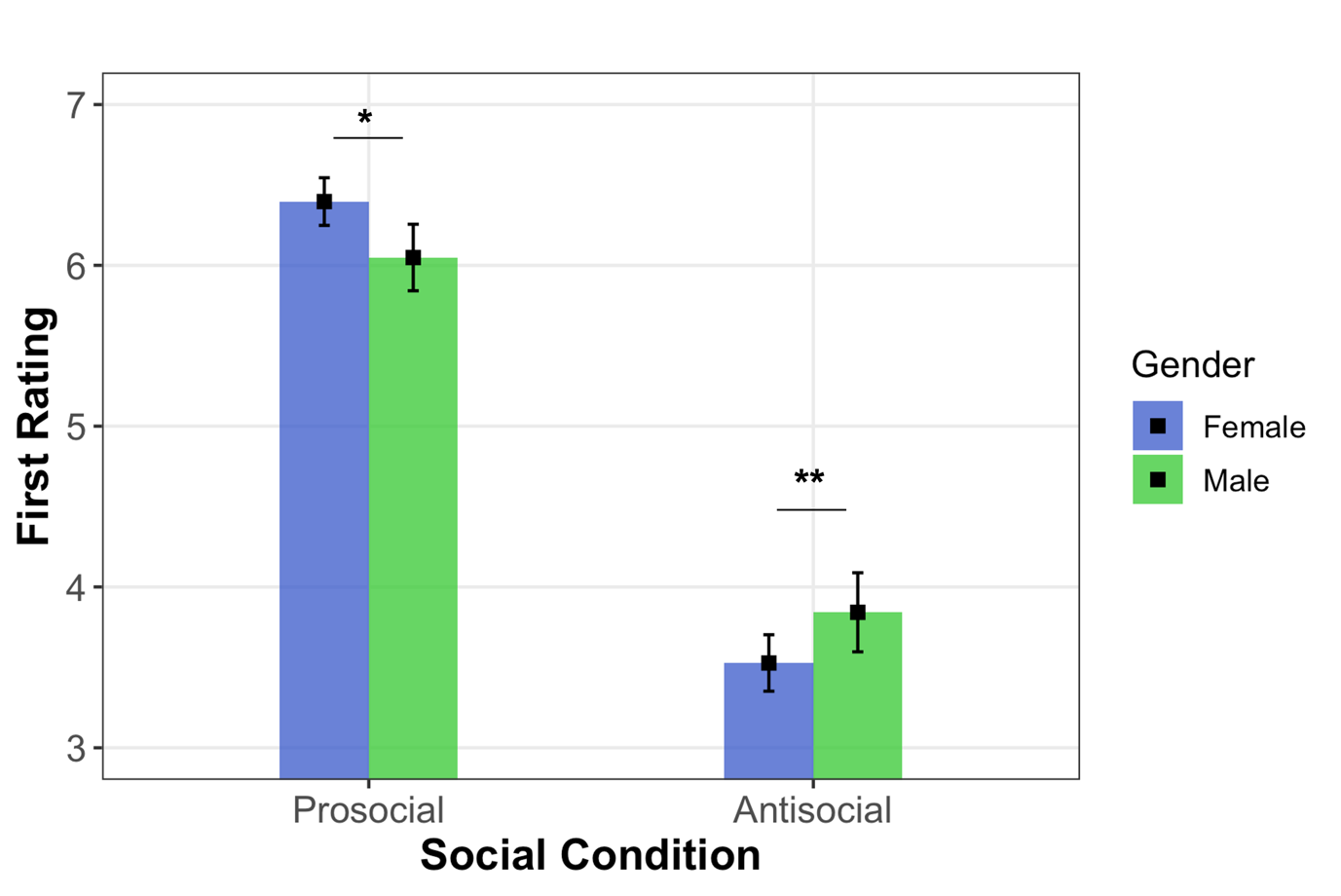


*Figure S2. Effect of Gender and Social Condition on First Ratings*

The figure shows mean first ratings of male (green) and female (blue) participants between social conditions. The squares show model predicted mean estimates of first ratings by gender and social condition and error bars represent corresponding 95% confidence intervals. Females show higher mean prosocial first ratings, as well as lower mean antisocial first ratings, than males. Asterisks indicate **p* < .05 ***p* < .01


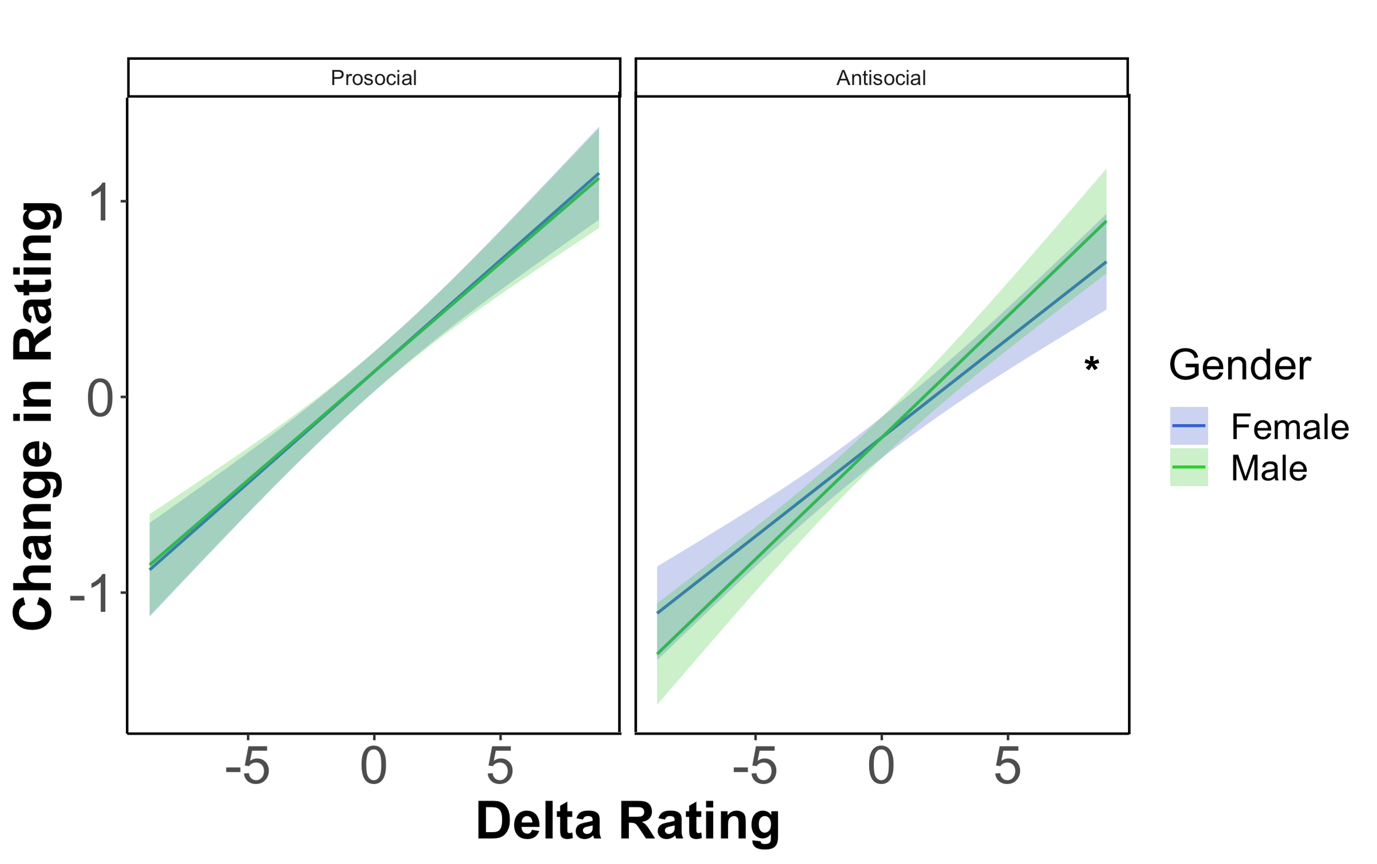


*Figure S3. Effect of Gender and Social Condition on Social Influence*

The figure shows differences in model predicted change in rating as a function of delta rating between males (green) and females (blue) and corresponding 95% confidence intervals (shaded area) in the prosocial (left panel) and antisocial (right panel) conditions. Female participants show less susceptibility to antisocial influence than males. Asterisks indicate **p* < .05


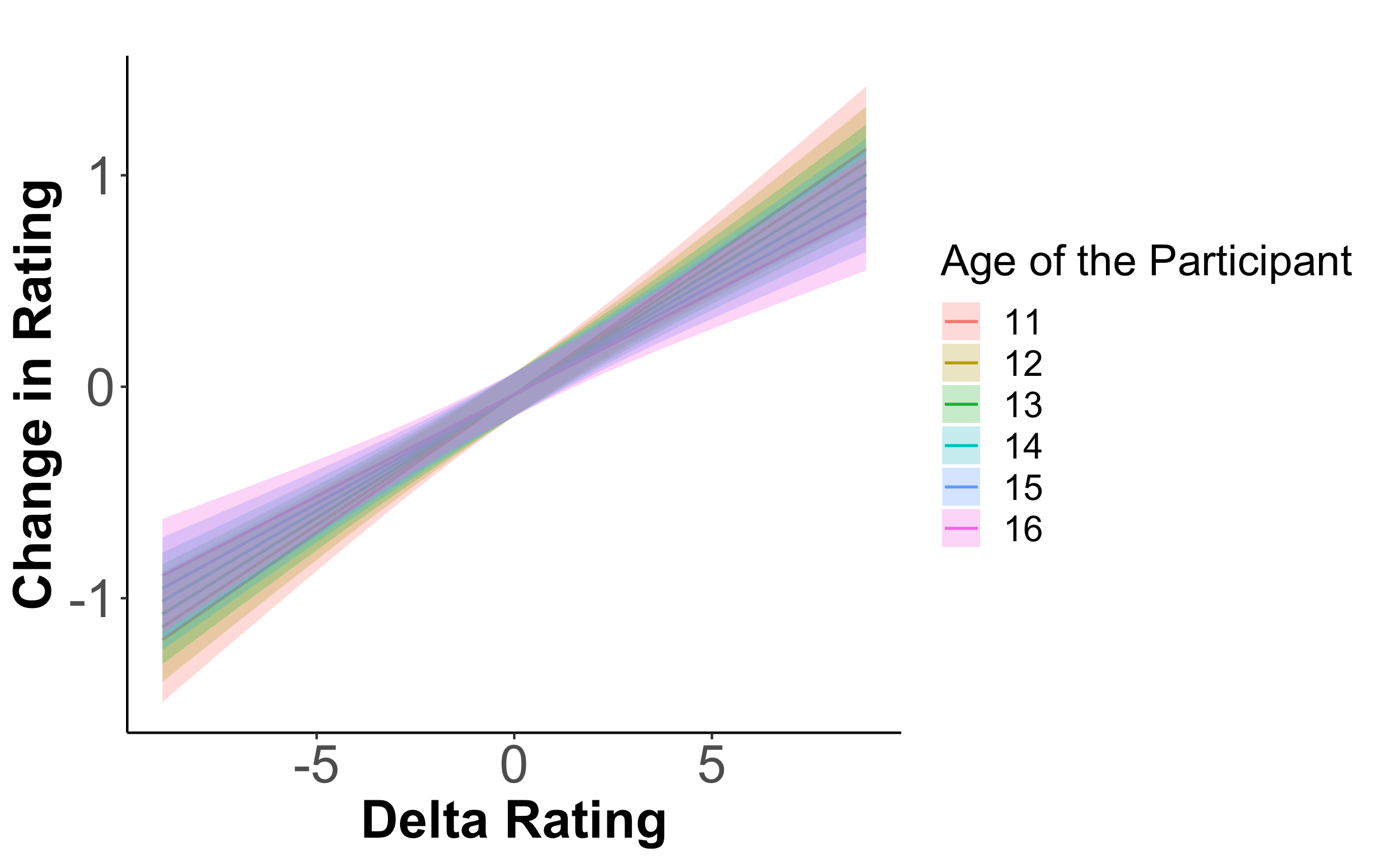


*Figure S4. Effect of Age on Social Influence*

The figure shows age differences in model predicted change in rating as a function of delta rating. Each colour represents an age group (11-16), and each line represents age-specific slopes of change in rating as a function of delta rating. Younger participants show a stronger effect of delta rating on change in rating, as indicated by a steeper slope (red, yellow, and green). As age progressively increases, the slope becomes flatter (blues and pink), indicating a smaller effect of delta rating on change in rating.


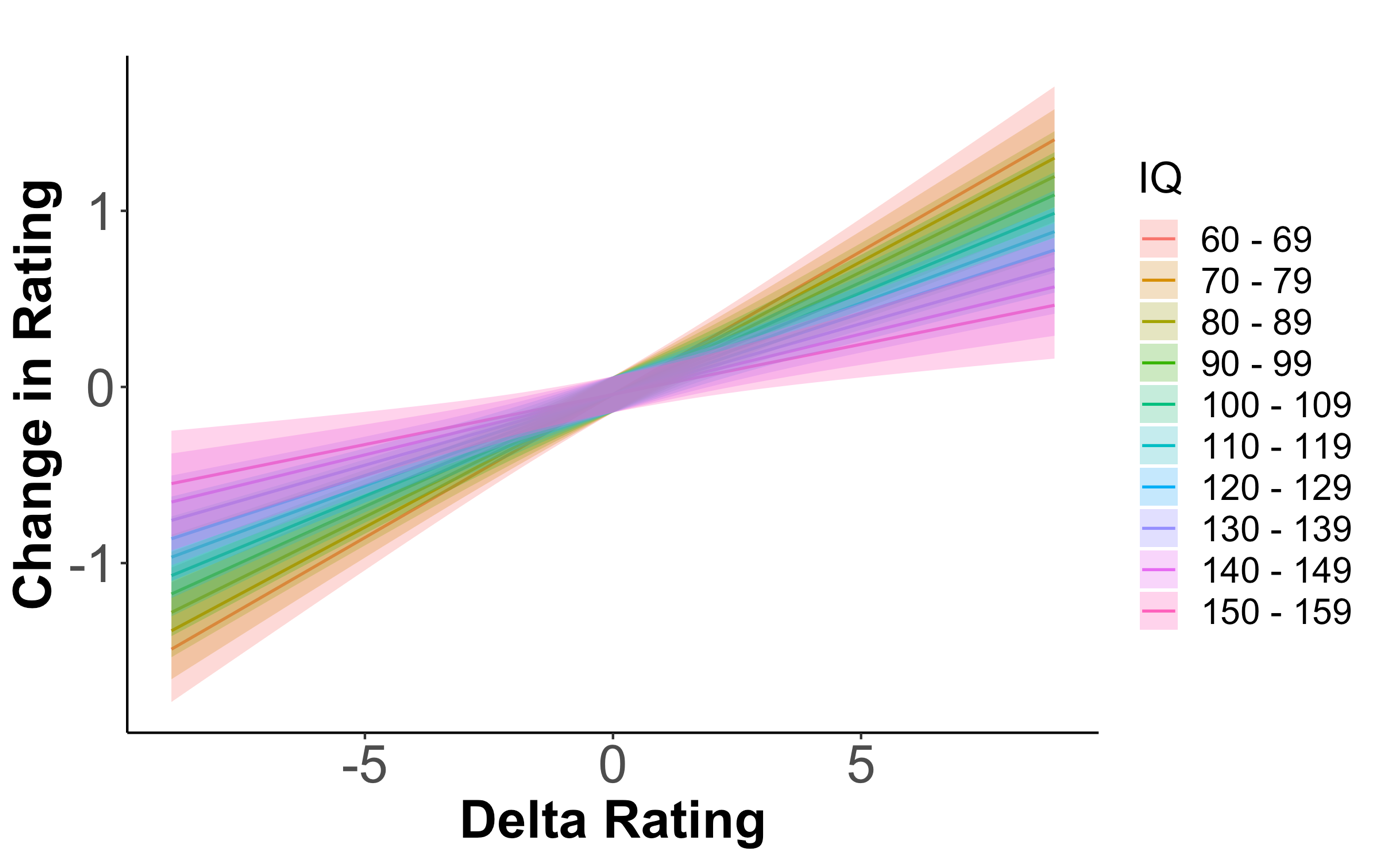


*Figure S5. Effect of IQ on Social Influence.*

The figure shows IQ differences in model predicted change in rating as a function of delta rating. Each colour represents an IQ interval (60-159; 10 intervals), and each line represents IQ interval-specific slopes of change in rating as a function of delta rating. Participants with a lower IQ show a stronger effect of delta rating on change in rating, as indicated by a steeper slope (red, orange, and yellow). As IQ progressively increases, the slope becomes flatter (purple and pinks), indicating a smaller effect of delta rating on change in rating.
